# A Convolutional Neural Networks-Based Approach for Texture Directionality Detection

**DOI:** 10.3390/s22020562

**Published:** 2022-01-12

**Authors:** Marcin Kociołek, Michał Kozłowski, Antonio Cardone

**Affiliations:** 1 Institute of Electronics, Lodz University of Technology, Al. Politechniki 10, 93-590 Łódź, Poland; 2 Department of Mechatronics, Faculty of Technical Science, University of Warmia and Mazury, Ul. Oczapowskiego 11, 10-710 Olsztyn, Poland; michal.kozlowski@uwm.edu.pl; 3 Information Technology Laboratory, Software and Systems Division, National Institute of Standards and Technology, 100 Bureau Drive, Gaithersburg, MD 20899, USA; antonio.cardone@nist.gov

**Keywords:** directionality detection, texture, convolutional neural networks

## Abstract

The perceived texture directionality is an important, not fully explored image characteristic. In many applications texture directionality detection is of fundamental importance. Several approaches have been proposed, such as the fast Fourier-based method. We recently proposed a method based on the interpolated grey-level co-occurrence matrix (iGLCM), robust to image blur and noise but slower than the Fourier-based method. Here we test the applicability of convolutional neural networks (CNNs) to texture directionality detection. To obtain the large amount of training data required, we built a training dataset consisting of synthetic textures with known directionality and varying perturbation levels. Subsequently, we defined and tested shallow and deep CNN architectures. We present the test results focusing on the CNN architectures and their robustness with respect to image perturbations. We identify the best performing CNN architecture, and compare it with the iGLCM, the Fourier and the local gradient orientation methods. We find that the accuracy of CNN is lower, yet comparable to the iGLCM, and it outperforms the other two methods. As expected, the CNN method shows the highest computing speed. Finally, we demonstrate the best performing CNN on real-life images. Visual analysis suggests that the learned patterns generalize to real-life image data. Hence, CNNs represent a promising approach for texture directionality detection, warranting further investigation.

## 1. Introduction

### 1.1. Texture Directionality Definition

Image texture carries valuable information about the spatial arrangement of intensity values in an image. It plays a fundamental role in image classification, and it can also be useful when performing image segmentation. A formal definition of texture can be given referring to its inherent structure, which generally consists of regularly repeating patterns. These patterns can be identified with respect to the smallest textural element, i.e., the texton [1] or the texel [2]. Such a structure-based texture definition is meaningful and can be especially useful when defining artificial textures. Alternatively, texture can be formally defined based on the characterization of the intensity arrangement in the image. Such a statistical approach is also meaningful and possibly more general, since natural textures are irregular, and it is not always possible to clearly identify structural patterns. Ultimately one can safely state that, due to the inherent complexity of image texture, attempts to formally define texture have not been completely successful so far. It is important to observe that texture directionality is a local property, not necessarily constant throughout the image.

**Figure 1:**
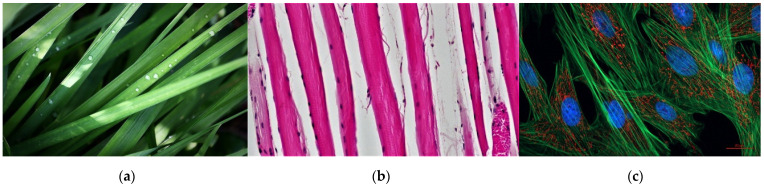
Instances of textures with clearly perceived directionality: (**a**) grass [3], (**b**) Muscle Tissue—Skeletal Muscle Fibers [4], (**c**) Indian Muntjac fibroblast cells [5].

Notwithstanding the lack of an agreed-upon formal definition for texture, both structure-based and statistical definitions suggest that texture direction is often clearly perceived by the observer. This is due, for instance, to local anisotropy as shown in Figure 1.

**Figure 2:**
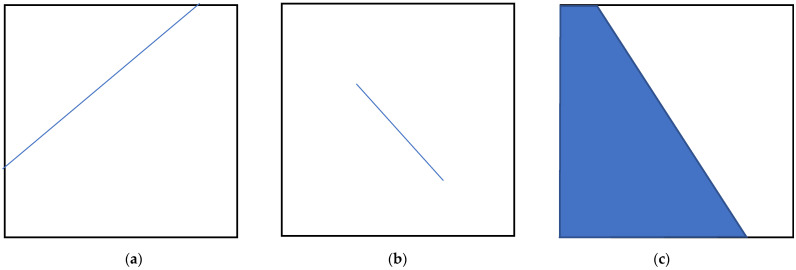
Entities determining texture directionality perception: (**a**) line; (**b**) segment; (**c**) edge.

Henceforth, we will refer to the clearly perceived direction of texture as texture directionality. Notably, texture directionality is closely related to the presence of entities such as lines, segments, or edges (Figure 2), which occur extensively in real-life applications.

**Figure 3:**
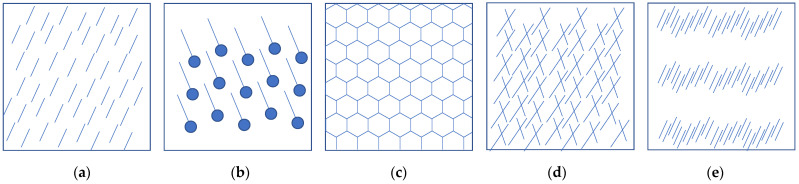
Texture directionality instances with different periodicity. (**a**) Simple line, periodicity 180°; (**b**) oriented line, periodicity 360°; (**c**) honeycomb, periodicity 120°; (**d**) multiple directionalities coexisting at the same scale; (**e**) multiple directionalities coexisting at different scales.

In this work, we focus on the quantitative characterization of texture directionality, arguably a texture property of great importance. The complexity of texture makes directionality detection extremely challenging. On one hand, depending on the specific entities leading to texture directionality perception in the image, directionality can carry different periodicity (Figure 3a–c). On the other hand, many directions can coexist in the same instance (Figure 3d). Furthermore, directions can be perceived at different scales, depending on the size of the entities determining texture directionality (Figure 3e).

With respect to texture periodicity, we limit ourselves to cases of strong local anisotropy for relatively small regions of interest (e.g., image tiles), for which one direction is present, with 180° periodicity.

From the computational perspective, texture analysis can be quite expensive due to the above-mentioned inherent complexity of texture. This is exacerbated by the need of terabyte-size texture analysis, often in real-time, for instance in fields such as microscopy, remote sensing, and astronomy [6,7]. Therefore, it is especially important to assess the computational efficiency of texture analysis techniques, and in particular of the texture directionality detection techniques addressed in this paper.

### 1.2. Related Work

Texture directionality analysis finds applications in many fields of scientific interest. In the image processing field, it plays an important role in image classification and retrieval, as shown in [8,9,10]. Texture directionality features have also been used for image coding [11].

In the biomedical field, texture directionality analysis can be extremely insightful into biological phenomena of interest. In [12], the directionality distribution of collagen fibers is associated with abnormal collagen morphology, a biomarker for several pathologies. In [13], texture directionality analysis is applied to the study of extracellular elastin and fibrillar collagen, whose directional arrangement is associated with atherosclerosis progression. In [14], breast carcinoma cells are shown to be extremely sensitive to the collagen direction and relative alignment.

In the material science field, texture directionality is associated with material properties. In [15], the orientation of carbon nanotubes (CNT) is correlated to properties of CNT-based materials such as strength and electric conductivity. Texture directionality is also used to characterize the magnetic particle alignment in photosensitive polymer resins [16]. Other applications include the astronomy field: in [17], an effort targeting high-throughput texture directionality analysis of solar images captured by the Solar Dynamic Observatory mission is described.

Several computational approaches for texture directionality analysis have been developed. The most widely used method is arguably the one based on the Fourier transform [18] and implemented as a plugin in Fiji/ImageJ software [19]. Additional approaches are based on Radon transform [20], Mojette transform [21], and Sobel filter. In [22], the authors use the Fiji/ImageJ local gradient orientation (LGO) method, based on Sobel filter, to characterize texture directionality in endothelial cells. Fractional Sobel filters provide enhanced flexibility, but to properly define the fractional order and other parameters can be challenging [23]. Other approaches for texture directionality analysis rely on the autocovariance function [24] or on the gray level co-occurrence transform (GLCM) [12,25]. GLCM features have been used for texture classification [26] and for the analysis of multispectral texture images [27], showing great potential for texture directionality analysis. In [28], the authors propose a texture directionality detection method relying on the optimization of an objective function, which involves the computation of GLCM features along four directions. Recently, we developed and implemented a texture directionality detection method using an interpolation-based version of GLCM (iGLCM), which can be computed along any direction [29]. The implementation of our iGLCM method is available in a Github repository [30]. Wavelets also have great potential for texture directionality analysis. They have been used successfully for image texture classification in the biomedical field [31] and in the manufacturing field [32]. Gabor wavelets, in particular, are closely related to structuredness and directionality of texture. They have been successfully used to characterize texture directionality in applications such as iris recognition, plant leaf recognition, and mammogram analysis [33]. An important challenge associated to wavelets is the proper definition of the right wavelet base and decomposition level to use [32,34].

Convolutional neural networks (CNNs) are becoming a leading image processing tool. They are extensively used for image classification, detection, and segmentation [35]. They have shown impressive performance on texture representation and classification [36,37,38], texture synthesis [39,40], as well as on texture anomaly detection [41]. In [42], the authors propose a training augmentation method for shape-texture debiased learning, successfully applied to texture recognition. In [43], CNNs are used to predict vegetation damage based on texture and other properties of tree images. Another interesting application of CNNs is described in [44], where the authors propose a deep learning-based cloud detection method with application to remote sensing. The deep network consists of a Gabor transform-based encoder-decoder, and of attention modules that enable the filtering of irrelevant image and texture information. Due to the scarcity of labeled data in many applications, transfer learning and data augmentation are used in many of the above papers. To our knowledge, CNNs have not been used for texture directionality detection. From a computational efficiency perspective, one can reasonably expect that CNNs will enable faster computations when targeting texture directionality detection, especially on machines equipped with GPUs (Graphical Processing Units) that allow for hardware-supported convolution operations. From a computational accuracy perspective, some studies report that CNNs are sensitive to textural features [45], suggesting they may perform well on texture directionality detection.

In the last decade, as a result of computer vision competitions such as the ImageNet Large Scale Visual Recognition Challenge (ILSVRC) [46], many widely used deep network architectures have been created, including AlexNet [47], VGG [48], and ResNet [49]. However, their size is considerable and results into a large number of parameters. Furthermore, some of these networks are designed to tackle specific issues, not necessarily relevant in every application. For instance, ResNet employs connections skipping to avoid the problem of vanishing gradients or to mitigate accuracy saturation [49].

### 1.3. Technical Approach

Texture directionality analysis is computationally intensive due to the inherent complexity of image texture and the need to characterize it locally. Furthermore, it can be sensitive to image noise and blur. The Fourier transform-based method implemented in Fiji/ImageJ [19] is fast and robust to image noise, but it can be extremely sensitive to image blur [29]. The iGLCM-based method, on the other hand, has comparable accuracy and is robust to both image blur and noise. However, due to the multiple required GLCM computations for a given region of interest, the iGLCM-based method is computationally intensive [29].

In this paper we propose an alternative approach for texture directionality detection based on CNNs. Both shallow and deep CNNs have the potential to accurately and efficiently detect texture directionality if properly designed and trained. The two main challenges associated with the use of CNNs are discussed below.

The first challenge is defining the CNN architecture. In general, depending on CNN size and features, a large number of parameters is involved in training and testing. Therefore, one should try to minimize the number of parameters without adversely affecting CNN performance. Given a specific application, rarely is there consensus on the optimal size and number of layers for a CNN [50]. Since using available CNNs is not always viable, it is common practice to investigate different CNN architectures empirically. One can start with shallower networks and gradually deepen them by increasing the size and number of layers, trying not to adversely affect accuracy and speed. This is the approach we employ in this work.

The second challenge is related to CNN training and testing. Generally, the amount of training data needed for CNNs can be overwhelming, especially when involving the tedious manual or semimanual annotation of images. In some cases, synthetic images with specific properties can be used for CNN training. Here we opt for the latter approach; to this end, we specifically define synthetic texture images with known texture directionality and perturbation levels for CNN training. On one hand, this will avoid the need for time-consuming manual annotations. On the other hand, the expectation is that CNNs will be able to learn texture directionality based on artificial texture images, and possibly generalize to real-life images.

## 2. Materials and Methods

### 2.1. Synthetic and Real Texture Images for CNN

We created a large set of synthetic images with well-defined texture directionality for CNN training and testing as follows. Initially, images with size 1000 × 1000 pixels consisting of evenly spaced vertical bars were created. By definition, each image is fully characterized by its bar thickness (e.g., 4 pixels) and period (defined as the distance between rising or falling edges of consecutive bars, in pixels), with the constraint that bar period should always be larger than bar thickness. A total of nine different bar thickness/period combinations were used: 2/8, 3/6, 4/8, 6/12, 8/16, 16/32, 20/40, 24/48, 32/64.

Each image was saved in 16-bit monochrome format, with background intensity of 16,384 (1/4 of the total range) and foreground intensity of 49,151 (3/4 of the total range). Subsequently, the image was subjected to an affine rotation of 180 different angles over the range (0°, 179°). After the rotation, the central part of the image (500 × 500 pixels) was cropped to remove blank areas. Each image was then saved, representing synthetic textures with no perturbation. A total of 9 × 180 = 1620 synthetic textures was obtained at this stage.

Furthermore, perturbations were applied to such synthetic textures to take into account the effect of noise and blur. The image perturbations consist of additive Gaussian noise with zero mean and four standard deviations values (2000, 4000, 6000 and 8000), as well as of blur averaging filters with four kernel sizes (3 × 3, 5 × 5, 7 × 7 and 9 × 9). After perturbations, the resulting set of synthetic textures amounts to a total of 1620 × (1 + 4 + 4) = 14,580 images.

**Figure 4:**
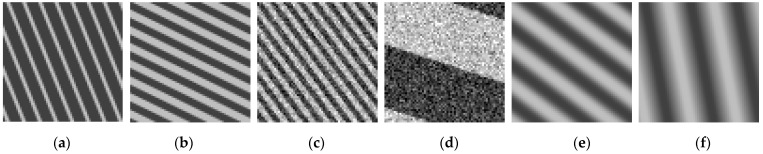
Instances of synthetic texture images. The directionality is reported with respect to vertical direction. (**a**) Bar thickness 2, bar period 8, directionality 22°, no perturbation; (**b**) bar thickness 4, bar period 8, directionality 64°, no perturbation; (**c**) bar thickness 3, bar period 6, directionality 37°, Gaussian noise with std equal 6000; (**d**) bar thickness 32, bar period 64, directionality 72°, Gaussian noise with std equal 8000; (**e**) bar thickness 8, bar period 16, directionality 53°, averaging blur with kernel 5 × 5; (**f**) bar thickness 12, bar period 24, directionality 11°, averaging blur with kernel 9 × 9.

Finally, the synthetic textures were divided into 49 nonoverlapping rectangular tiles of size 64 × 64 pixels. The final set of synthetic textures amounts to 14,580 × 49 = 714,420 images. This set was shuffled and split into three subsets. The first subset amounts to half of the images (357,210) and is used as training set. The second and third subsets amount to one quarter of the images each (178,605) and are used as validation and testing sets. Figure 4 shows instances of synthetic texture images.

The above procedure for building synthetic textures was implemented using the Python module CV2 for computer vision, a library of Python bindings with OpenCV (Open Source Computer Vision) [51]. The obtained images and metadata (i.e., texture direction, perturbation level) were stored using Pickle, a user-friendly Python module for serializing and de-serializing objects and data structures [52].

In order to demonstrate the performance of CNNs on real-life image data, we also employed a limited set of texture images from various sources, such as the Brodatz textures [53] and cell images obtained at the National Institute of Standards and Technology (NIST) [54].

### 2.2. CNN-Based Directionality Detection

Texture directionality detection using CNNs can be addressed as a regression or a classification problem. In the first case, the CNN will predict one direction for a given texture image. However, as discussed in Section 1.1, in many cases several directions coexist in a texture. Each direction can in general be perceived differently, e.g., more or less clearly. Therefore, ideally one would like a texture directionality detection tool that can detect one or more directions, and the relative importance of the various directions. Hence, in this work, we address CNN-based texture directionality detection as a classification problem.

The output for a classification problem is generally a tensor of probabilities, whose size is problem dependent. In our case, the output size is related to the chosen resolution, since each component of the probability tensor represents a directionality angle. We use CNN architectures with output of size 180, corresponding to a 1° resolution for a directionality range ⟨0°,179°⟩. The resulting probability values are used to select texture directionality as the tensor component associated to the maximum value. However, in some cases (e.g., homogeneous images) the maximum probability value might not represent a meaningful texture direction. In such cases, the associated directionality should be discarded. We propose to discard the direction associated to the maximum probability value, if that value is smaller than a given probability threshold. The probability threshold can be found empirically, and it depends on the image data to analyze. In our case, tests on our image data led to a probability threshold value equal to 0.011, around twice as much as the mean probability value (i.e., 1/180).

To select a CNN architecture, as discussed in Section 1.3, our proposed approach is to empirically define and test shallow and deeper architectures, in an attempt to identify the best performing ones with limited number of parameters. Hence, we designed twelve CNNs, four shallow and eight deep. For the design, training and testing of the CNNs we used Keras [55], an open-source software based on the TensorFlow library and with a Python interface [56] (version 3.6.12 of Python, 1.15.4 of TensorFlow and 2.2.4-tf of the module TensorFlow-Keras, a TensorFlow-specific implementation of the Keras API). The CNNs are described in Table 1, Table 2 and Table 3 and in Figure S1 from the Supplementary Materials.

The shallow CNNs (SN1-SN4) consist of one convolutional layer followed by global max pool, dropout, and output layer (Table 1). The global max pool layer makes the CNN independent of the input image size to some extent, unlike many available networks [47,48,49]. Basically, the minimum allowed input image size is limited by the size of the convolutional layer filter. The number of filters in the convolutional layer is equal to the output size for CNNs SN1 and SN3, and to half of the output size for CNNs SN2 and SN4. Two filter sizes were used for the convolutional layer: 17 × 17 for CNNs SN1, SN2 and 13 × 13 for CNNs SN3, SN4.

The eight deep CNNs (DN1-DN8) consist of three convolutional layers: the first two (layers 2 and 5) are followed by max pool and dropout layers, the last one (layer 8) is followed by global max pool and dropout (Table 2 and Table 3). Then, a fully connected layer (# 11) is followed by dropout and by the output layer. Akin to the shallow CNNs, the global max pool subsampling layer makes the networks independent of the input image size to some extent. In this case, the minimum allowed input image size is given by Equation (1).

(1) $$minImSize=\left(cf_{8}\cdot mp_{6}+\left(cf_{5}-1\right)\right)mp_{3}+\left(cf_{2}-1\right)$$

where:

$cf_{k}\;$= size of convolutional filter at layer *k*,

$mp_{k}\;$= size of max pool subsampling at layer *k*

For the first convolutional layer, the filter kernels sizes are 17 × 17 (DN1 to DN4) or 7 × 7 (DN5 to DN8). For the remaining two convolutional layers, the filter kernels sizes are 5 × 5 and 3 × 3. Subsequently, the minimum input image size is 36 × 36 pixels for the deep networks DN1 to DN4, and 26 × 26 for the deep networks DN5 to DN8. Please note that the minimum input image sizes for deep networks are larger than in the case of shallow networks.

The number of filters in the first convolutional layer is equal to 16 for CNNs DN1, DN2, DN5 and DN6, to 90 for CNNs DN3, DN4, DN7 and DN8. The number of filters in the second convolutional layer is equal to 16 for CNNs DN1, DN3, DN5 and DN7, and to 32 for CNNs DN2, DN4, DN6 and DN8. The number of filters in the third convolutional layer is equal to 90 for all deep CNNs

It is worth mentioning that all convolutional layers employ filters with no padding. This is because padding will change the local direction at the edge of the image, hence creating an artifact that will likely affect the accuracy of directionality prediction. The SM (soft max) activation function is always used in the output layer. For all other convolutional and dense (fully connected) layers seven different activation functions were tested: ReLU (rectified linear units), ELU (exponential linear units), SELU (scaled exponential linear units), Si (sigmoid), SP (soft plus), SS (soft sign) and TanH (hyperbolical tangent). They are shown in Figure S2 of Supplementary Materials [57].

The activation functions have different properties. Some of the activation functions are not differentiable at zero, nonlinear and/or bounded for negative or positive values. They can be symmetrical with respect to zero. The expectation is that the performance of a CNN on directionality detection will be affected by some of the properties of the activation functions.

### 2.3. Training and Testing Procedures

Training was performed on the Enki cluster at NIST, consisting of IBM Power System AC922 (IBM, Armonok, NY, USA) compute nodes equipped with 575 GB DDR4 memory, two IBM POWER9 SMT4 Monza 20-core CPUs and four Nvidia Tesla V100 SXM2 GPUs. On the other hand, testing was performed on a PC running with Ubuntu 20.4 OS (Canonical Ltd. London, UK) and equipped with an Intel Core i7-9800X (8 cores, 16 threads, 3.8 GHz) CPU (Intel, Santa Clara, CA, USA), a Nvidia Titan RTX (4608 CUDA cores with 24GB of DDR6 RAM) GPU (Nvidia, Santa Clara, CA, USA), and 128 GB of RAM. The following training procedure was carried out for each of the 84 combinations of the twelve CNNs and seven activation functions. For each combination, three training replicates were carried out starting from randomly set CNN parameters. Training was carried out on batches of 32 input images for a total of 200 epochs. The loss function we used is based on the categorical cross-entropy, which is a customary choice since in this work we address directionality detection as a classification problem. The formal definition of the loss function is provided as follows [58].

(2) $$loss=-\frac{1}{N}\overset{N}{\underset{i=1}{\sum }}\overset{C}{\underset{j=1}{\sum }}t_{j}\left(x_{i}\right)\log p_{j}\left(x_{i}\right)$$

In Equation (2) N is the number of observations, $x_{i}$ represents the general observation, C is the number of classes (in our case equal to 180, the number of directions considered), and $t_{j}\left(x_{i}\right)$ is the j-th element of the one-hot encoded label for the observation $x_{i}$ with the following conditions: $t_{j}\left(x_{i}\right)\in \left\{0,1\right\}$ and $\overset{c}{\underset{j=1}{\sum }}t_{j}\left(x_{i}\right)=1\;\forall \;i$. Furthermore, $p_{j}\left(x_{i}\right)$ is the j-th element of the network output (i.e., prediction) for the observation $x_{i}$. Since the output layer activation function is SM, the following conditions hold: $p_{j}\left(x_{i}\right)\geq 0$ and $\overset{c}{\underset{j=1}{\sum }}p_{j}\left(x_{i}\right)=1\;\forall \;i$. The element $p_{j}\left(x_{i}\right)$ represents the probability that the observation $x_{i}$ belongs to the class j. The resulting loss value is unitless.

The same synthetic texture image set, consisting of 357,210 training images and 178,605 validation images, was used for all training. For each replicate, training and validation curves were used for evaluation.

The remaining 178,605 synthetic texture images, consisting of a different set from the training and validation images, were used for testing. The accuracy of texture directionality detection was computed across different bar sizes and perturbation levels (i.e., noise standard deviation values and blur kernel sizes). The categorial cross-entropy is not the best choice for testing purposes, since by definition it does not take into account angle periodicity, and it is not proportional to the angle error. Therefore, we used the directionality prediction error defined in Equation (3), which accounts for 180° periodicity:

(3) error=acos(|cos(α−β)|)

where:

α—true direction,

β—predicted direction.

## 3. Results

### 3.1. Training and Testing on Synthetic Textures

**Figure 5:**
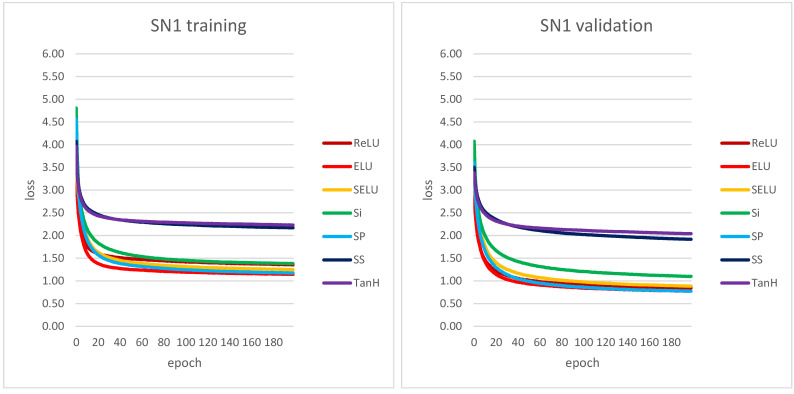

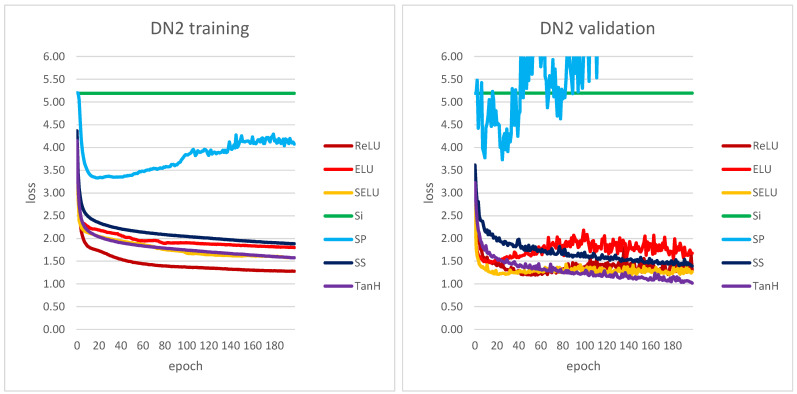
Training and validation loss curves for the best performing shallow and deep CNNs (best performing replicate). For DN2 validation plot, the SP plot was clipped due to its large fluctuations to keep the most appropriate plot scale. For the loss function, please refer to Equation (2) and related text.

The twelve CNN architectures described above were initially evaluated using the training and validation curves obtained from Keras libraries and functions [55]. The three training replicates were consistent with no major differences (see Figures S3–S9 in Supplementary Materials). Figure 5 shows the learning curves for the best performing shallow and deep CNNs, SN1 and DN2. Figure 5 also shows the training replicate with the best performance.

For shallow CNNs, the majority of training and validation loss plots are smooth and show asymptotic behavior within 200 epochs. Most of the plots decrease monotonically, but for CNNs/activation function pairs SN3-ELU, SN4-ELU, SN2-SELU, SN3-SELU, SN4-SELU, SN3-SP and SN4-SP, the training loss functions reach a minimum between epochs 50 and 80 and then slightly increase (see Supplementary Materials Figures S10–S13). The best performing CNN is SN1, the network with the largest number of input filters (180) with the largest size (17 × 17). For SN1, the best performances occur with the activation functions ELU, SP, ReLU and SELU, whereas the worse performances occur with TanH and SS. Based on our data, the decreasing of number and size of input filters seem to generally lead to a decrease in CNN performance.

For deep CNNs, the behavior of training and validation loss curves was quite different (see Supplementary Materials Figures S14–S21). In the case of the Si and SP activation functions, the training was not successful for any of the deep CNNs. For Si the learning loss curve does not decrease over the 200 epochs, whereas for SP it shows extremely large fluctuations. For the remaining activation functions, the validation loss curve consistently shows smaller fluctuations around smaller values, still suggesting possible instability in some cases. Overall, the best performing deep CNN is DN2. For CNNs DN2, DN3, DN4, DN6, DN7 and DN8, the TanH activation function is associated with the lowest validation loss and shows relatively small fluctuations throughout. Similarly, the SS activation function shows relatively small fluctuations, but the validation loss is higher than TanH. Overall, deep CNNs with larger kernels in the first convolutional layer show lower values in both training and validation loss (see Supplementary Materials Figures S14–S21).

**Figure 6:**
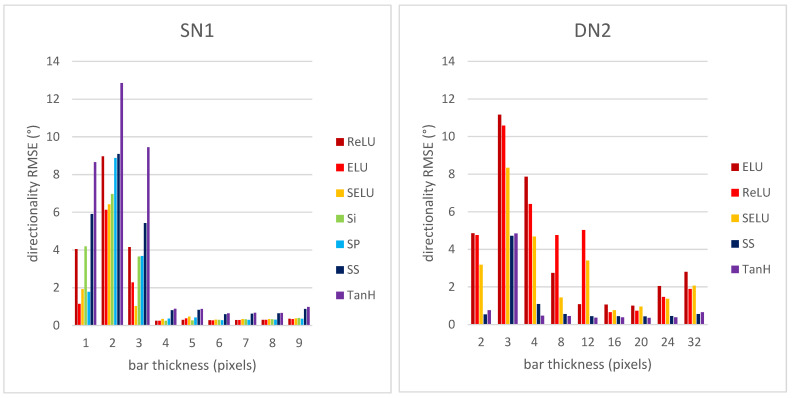
Directionality RMSE for CNNs SN1 and DN2 vs. synthetic texture bar thickness.

Figure 6 shows a bar plot of the directionality RMSE using the error from Equation (3) vs. bar thickness for CNNs SN1 and DN2.

The best activation functions for the shallow CNN SN1 are ELU and SELU, whose training and validation also lead to low loss values. The worse activation function for the CNN SN1 is TanH. On the other hand, for the deep CNN DN2, the best activation functions are TanH and SS. TanH shows the best performance based on the training loss curve as well. For both shallow and deep CNNs, the largest RMSE values correspond to synthetic textures consisting of narrow bars (i.e., 2, 3, and 4 pixels wide). This is due to the effect of distortion (noise and blur), more noticeable on narrower bars, since the RMSE values are obtained over all distortion levels. Please note that for the deep CNN DN2, the training for the Si and SP activation functions was not successful, and hence the corresponding plots are not shown.

**Figure 7:**
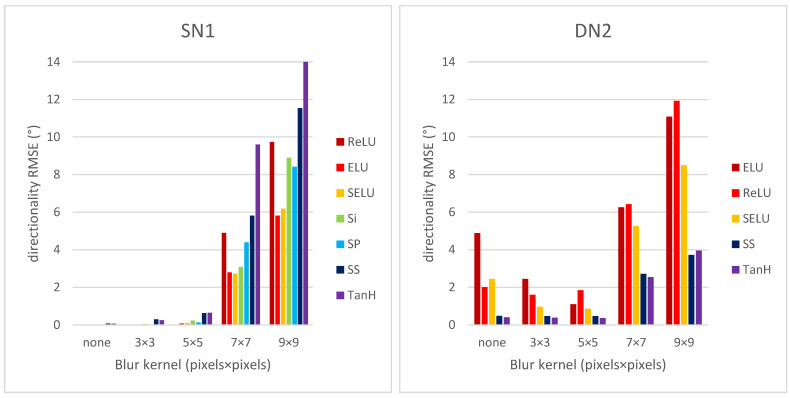
Directionality RMSE for CNNs SN1 and DN2 vs. blur kernel size.

Figure 7 shows the directionality RMSE vs. blur kernel size for CNNs SN1 and DN2.

Both shallow and deep CNNs are sensitive to blurring for kernel size 7 × 7 and beyond. As before, the best performing activation functions for the shallow CNN are ELU and SELU, whereas for the deep CNN are TanH and SS.

**Figure 8:**
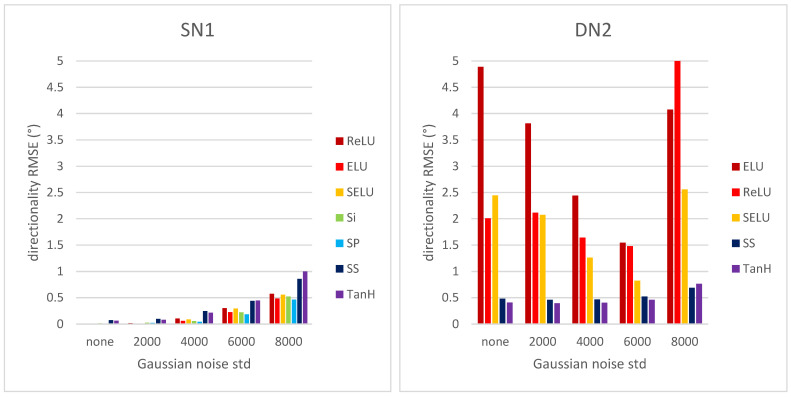
Directionality RMSE for CNNs SN1 and DN2 vs. Gaussian noise standard deviation.

Finally, in Figure 8 the directionality RMSE vs. Gaussian noise standard deviation for CNNs SN1 and DN2 is shown.

The shallow CNN SN1 seems robust to Gaussian noise for all activation functions as RMSE does not increase significantly with noise standard deviation levels. The deep CNN DN2 shows similar behavior only for the activation functions TanH and SP, whereas the remaining activation functions are more sensitive to noise, showing significantly higher RMSE values. Overall, deep CNNs with larger kernels in the first convolutional layers show a slightly better performance than deep CNNs with smaller kernels (see Supplementary Materials Figures S24 and S25).

The results presented above indicate CNN-based texture directionality detection shows good performance on synthetic texture data. The best performing shallow and deep CNNs are SN1 with the activation function ELU (henceforth called SN1-ELU) and DN2 with the activation function TanH (henceforth called DN2-TanH). They show comparable accuracy and computational efficiency, with SN1-ELU slightly outperforming DN2-TanH for all Gaussian noise levels and for small averaging blur kernels, up to size 5 × 5. On the other hand, for larger blur levels DN2-TanH outperforms SN1-ELU (please see Table S1 in the Supplementary Materials).

We used the shallow SN1-ELU network to assess the performance of CNN-based texture directionality detection with respect to more traditional techniques. We compared SN1-ELU with our iGLCM method [29] as well as with the Fourier and the LGO methods, both implemented in Fiji/ImageJ [18,19,22,23]. Table 4 shows the directionality detection RMSE values for the SN1-ELU and the three other methods.

The data were obtained on the same test dataset as above. RMSE values were obtained across different synthetic textures, for all blur kernel sizes and Gaussian noise standard deviation levels used earlier. Generally, the SN1-ELU method has a slightly worse performance, but comparable to the iGLCM. In particular, the iGLCM method slightly outperforms the CNN with respect to Gaussian noise, and the blur up to averaging filter size 5 × 5. Both the SN1-ELU and iGLCM methods outperform the Fourier and LGO methods, except for the highest blur perturbation value.

As expected, the computational gain achieved with the CNN method is considerable (~200 folds with respect to iGLCM, ~7 folds with respect to Fourier and LGO). The computations were performed on the PC whose specifications were reported at the beginning of Section 2.3.

### 3.2. Demonstration on Non-Synthetic Textures

The above results were obtained on synthetic image textures with known properties (i.e., direction and perturbation level). CNNs trained on synthetic image textures seem to enable texture directionality detection on other synthetic image textures, demonstrating the ability to generalize.

**Figure 9:**
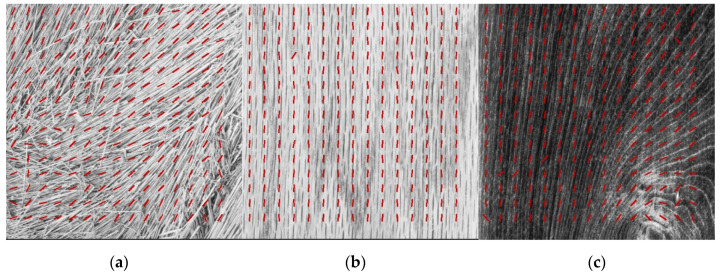

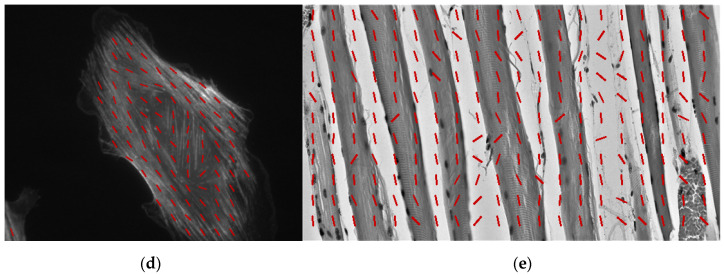
CNN-based directionality detection on: (**a**–**c**) Brodatz textures [53] obtained from publicly available image databases [59,60]; (**d**) microcopy images of actin-stained fibroblast cells, (**e**) Muscle Tissue: Skeletal Muscle Fibers [4].

Here, we test the directionality detection performance of CNNs on non-synthetic, real-life images, to further test the generalization capability of CNNs for texture directionality detection. To this end, we use selected images from Brodatz textures, a collection of grayscale texture photographs obtained by Phil Brodatz [53] and publicly available in image databases [59,60], as well as microscopy cell images showing protein filaments obtained at NIST [54]. The best performing CNN SN1 with ELU activation function was used for the purpose. Figure 9 shows representative results of CNN-based texture directionality detection.

Each image is tiled into rectangular tiles of size 64 × 64, equally spaced at 64 pixels, and texture directionality is computed for each tile (represented by red lines superimposed on the image). The assessment in this case can only be qualitative, since there is no reference data available. However, these results and additional tests (see Supplementary Materials Figure S22) clearly show that CNNs trained on synthetic data can perform well on real-life images with applications such as cell biology. For instance, the directionality of protein actin fibers within a fibroblast cell can be quantified using our CNN-based technique (Figure 9d), hence providing insight into the cell response to mechanical cues from the extracellular matrix [54]. It should be noted that for Figure 9d and Figure S22 (from Supplementary Materials), the probability threshold discussed in Section 2.2 was set so that no directionality is detected in the artificial extracellular matrix on which fibroblast cells were cultured. It is also important to observe that, in many applications, the tile-based directionality assessment might need to be combined into a macro textural feature (e.g., computed for the whole image or for specific regions of interest (ROI), consisting of groups of tiles) to study application-specific aspects. In some cases, texture analysis might also involve overlapping tiles for enhanced accuracy. To that effect, we show the polar histograms obtained for the Brodatz textures in Supplementary Materials Figure S23 to reinforce that our approach yields tile-based directionality assessment that is available to the user, and which can be combined into a macro textural feature if needed. In addition, data from the polar histograms can be used to compute higher order directionality features, such as the dominant direction and associated spread.

## 4. Discussion and Future Work Directions

In this paper, we studied the performance of CNN architectures of different size on texture directionality detection. We tested seven commonly used activation functions with each of the CNN architectures to fully characterize their performance. We carried out training and testing using synthetic texture images with varying perturbation levels to assess the robustness of CNN-based texture directionality detection.

Data suggests that asymmetrical and unbounded activation functions such as ELU and SELU have the highest accuracy for shallow CNNs. On the other hand, symmetrical and bounded activation functions such as TanH and SS seem to work better for deep CNNs. In general, shallow CNNs tend to outperform deep CNNs as far as robustness to noise and to lower blur levels. For the two highest blur levels, deep CNNs are slightly better than shallow ones (Figure S25 and Table S1 in Supplementary Materials).

**Figure 10:**
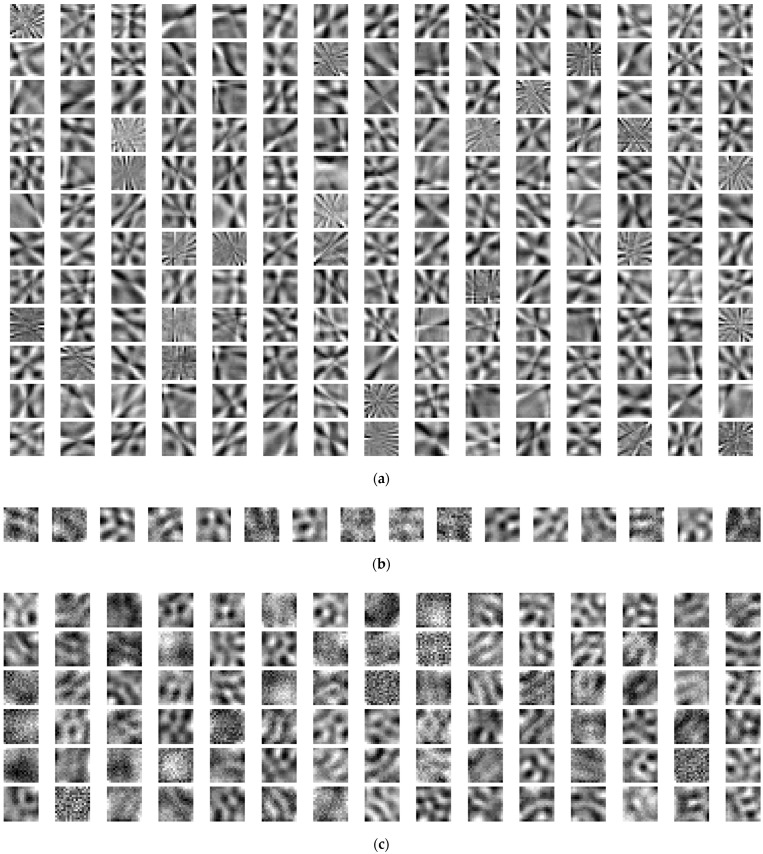
Filters from the only convolutional layer of the best shallow CNN, SN1-SELU ((**a**), 180 filters), from the first convolutional layer of the best deep CNN, DN2-TanH ((**b**), 16 filters) and from the first convolutional layer of the deep CNN, DN4-SS ((**c**), 90 filters). The filters are shown for one training instance.

In order to gain additional insight into the inner workings of shallow and deep CNNs, the filters belonging to the only convolutional layer of the best performing shallow CNN, SN1-ELU, and the filters belonging to the first convolutional layer of the best performing deep CNNs, DN2-TanH and DN4-SS, are shown in Figure 10 (see also Figures S26 and S27 in Supplementary Materials).

In general, the presence of noisy convolutional filters indicates that the training has not been fully successful [61]. In the case of SN1-ELU, none of the 180 filters is noisy and a directional component can always be discerned. The filters show varying frequency levels, and in most cases multiple directionalities coexist within the same filter. In the case of DN2-TanH and DN4-SS, quite a few of the filters are noisy in the first convolutional layer, and none of them has a clear directional component. Overall, the convolutional layer of the shallow SN1-ELU seems to enable the full characterization of texture directionality, as one would expect. On the other hand, the presence of noisy filters in the first convolutional layer of the DN2-TanH and DN4-SS suggests that texture directionality characterization does not fully occur in that layer, but subsequent layers may play an important role as well. Additional evidence to that effect comes from Figures S24 and S25 (in Supplementary Materials), where DN2-TanH, DN4-SS, DN6-TanH and DN8-TanH show better performance than DN1-TanH, DN3-SS, DN5-TanH and DN7-TanH, respectively. Notably, one important difference between the former and the latter deep networks is the size of the second convolutional layer (Table 2 and Table 3), suggesting such layer may play an important role in the performance of the deep CNN. We show filters from the first, second and third convolutional layer of DN2-TanH in Figure S27 (in Supplementary Materials). Once again, visual inspection suggests that a few of the 16 filters from the first layer are noisy, and none of the smaller filters from the second and the third layer seem to carry a clear directional component. An important related observation is that, in the case of deep networks, the directional information is not stored in a specific layer, but it lies across the convolutional layers.

Two main conclusions follow from above: (1) the performance of a CNN on texture directionality detection is closely related to the properties of the activation functions used; (2) based on our data, both shallow and deep CNNs show potential for texture directionality detection and warrant further investigation.

An important topic of discussion concerns the kernel sizes of the convolutional layers in the deep networks. The general tendency is to use smaller kernel sizes for the sake of computational efficiency, which for most applications does not adversely affect the accuracy of the network. However, in the case of texture directionality detection we believe larger filter kernels are more effective, especially when dealing with complex texture patterns. In fact, larger kernels enable a better correlation of the kernel parameters with such complex patterns, due to the higher number of parameters available. Indeed, larger kernels better fit to the texture “building block” (i.e., texton [1] or texel [2]), which is generally large in the analyzed textures. This results in more accurate representation of texture directionality by the deep network. In Figure S28a,b of the Supplementary Materials, two images of textures are shown. They are obtained using the same procedure that was used to generate synthetic textures (Section 2.1), and each of them contains one bar with a specific orientation (156° and 157°). Such orientations yield a complex texture, consisting of many intensity values due to the sampling process. This is an instance of textures whose difference in directionality is difficult to detect. As pointed out above, only the larger kernels will be able to distinguish such textures due to the possibility of a better correlation of the kernel with the complex texture structure. In Figure S28c of the Supplementary Materials another instance of a complex texture is shown, this time from a real-life image of a fibroblast cell [54]. In general, complex textures are present in both real-life image data and synthetic images.

In Figures S29 and S30 of the Supplementary Materials instances of the filtering outcome are shown for synthetic images with complex texture directionality. In this case, texture complexity is the result of image perturbation and/or specific bar orientation. The filtering was carried out using the 16 large (17 × 17) and small (7 × 7) kernels followed by the TanH activation function taken from the DN2-TanH and DN6-TanH networks. For each synthetic image, the 16 filtered instances are shown. Clearly, the filtering outcome from large kernels shows a stronger contrast with respect to small kernels. The contrast is quantified on the right of each filtered image row using the standard deviation and the intensity range. A stronger contrast represents a stronger signal resulting from the filtering, which implies a better preservation of the directionality component in the case of large kernels. Furthermore, in Table S2 of the Supplementary Materials the performance of deep networks DN2-TanH and DN6-TanH, whose only difference is in the size of the first filter kernel (17 × 17 and 7 × 7, respectively), are directly compared with focus on image perturbation. The data clearly show that the network with the larger kernel consistently outperforms the other one across all noise and blur levels. This is not extremely unusual for deep networks. A few instances of networks whose performance does not improve when using smaller filter kernels can be found in the literature, as well as studies focusing on the most appropriate kernel size selection to maximize the network accuracy [62,63,64,65].

In future studies we will assess the role of non-linearities that are present in the deep network layers. Our intuition is that, for the tested deep networks, most of the directionality information lies in the first layer. For instance, the first layer of the deep network DN2-TanH seems to be overwhelmingly more directional and less noisy than the remaining two by visual inspection (please see Figure S27 of the Supplementary Materials). This might be due to the presence of non-linearities such as the layer activation functions and pooling operations, which somehow prevent the full propagation of directionality information to the subsequent layers. We believe targeted modifications of the deep networks to eliminate the non-linear components should be investigated in the future, in order to assess if the directionality information can better propagate to the subsequent layers. That, in turn, might change the behavior of the network, especially with respect to the size of the first layer kernel.

Another important observation is related to the fact that shallow networks allow for smaller input tiles (e.g., SN1 is capable processing tiles of size 17 × 17) unlike deep networks (the minimum allowed tile size is 36 × 36 or 26 × 26 for the deep networks studied in this paper), as observed earlier. This is clearly an important feature of the network, since smaller tile size allows the analysis of smaller texture regions, and texture is by definition a local property. Therefore, this feature might make shallow networks more appealing for some applications.

Since there is room for improvement, several future research directions can be pursued. For instance, custom loss functions could be designed to explicitly take direction periodicity into account, thus possibly enhancing the performance of CNNs. An additional above-related research direction is the investigation of additional CNN architectures. This includes the design and testing of CNNs that will closely mimic directional filters for enhanced accuracy. For instance, one can investigate the performance of CNN architectures consisting of conveniently initialized and possibly partially constrained first convolutional layers, aimed at detecting specific texture directions. An important caveat is that the definition of novel CNN architectures always involves a trade-off between performance and generality or efficiency. According to our data, for both shallow and deep CNNs the directionality detection performance seems to generally increase with the size of the network. However, larger CNNs are computationally expensive and large number of convolutional layers enables the analysis of large images, hence adversely affecting the generality of the analysis.

Another future work direction is related to the criterion to discard texture directionalities when not meaningful (e.g., in the case of homogeneous images). As discussed in Section 2.2, we currently use an empirically found threshold value for the maximum probability, below which to discard the associated directionality. However, we believe that this is a limitation of our current approach, since threshold-based approaches are not general, and they usually depend on the analyzed image data. Therefore, more general approaches to properly handle homogeneous images should be pursued, not necessarily threshold-based. Furthermore, such approaches should also address the coexistence of multiple directions in a texture image.

**Figure 11:**
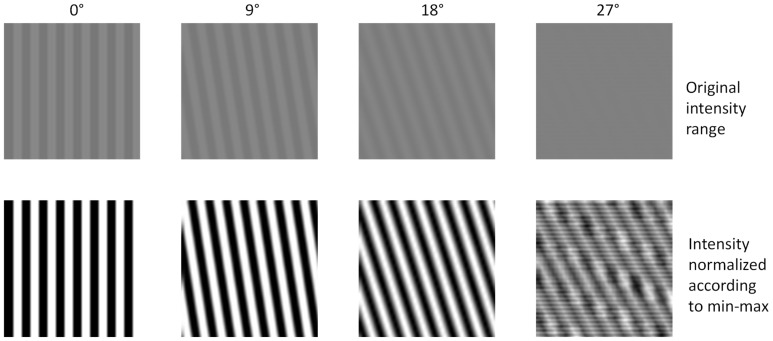
Instance of bars of bar size 4, bar period 8 and blurred by averaging filter with kernel size of 9 × 9.

An aspect that also warrants further analysis is the generally low performance of the proposed CNNs on synthetic texture images consisting of small bars perturbed using large blur kernels. An initial assessment of such cases based on visual inspection suggests that synthetic texture images can change their directionality properties when subjected to high blurring perturbations. Figure 11 shows synthetic textures with different directions and subjected to the highest tested blurring level.

At direction 27°, one can clearly notice the emergence of additional directionality patterns due to aliasing effects. In such case, the lower accuracy of CNNs is just an artifact. Related future research directions include more extensive testing, involving a broader range of synthetic data (e.g., more perturbation levels and different types or sizes). Such tests should, in particular, target cases as the one discussed above and shown in Figure 11, so that potential artifacts are eliminated or ruled out.

From the computational efficiency perspective, the best performing CNN network, SN1-ELU, was compared to the iGLCM, Fourier and LGO methods. The CNN method, which was implemented taking advantage of GPU acceleration, is the most efficient. This was surely expected when compared to the iGLCM method, which requires a total of *D*x*L* iGLCM computations, each involving O(*N*) expensive memory access operations (*D*, *L* and *N* are the number of directions used, number of offsets used, and number of pixels in the region of interest, respectively) [29]. It is worth mentioning that the currently implemented version of all methods can be further optimized (e.g., for GPU computing), hence potentially improving the reported efficiencies.

A final observation is that the best performing CNN network (SN1-ELU) was demonstrated using real-life images from various sources. While the analysis was purely qualitative, visual inspection suggests that carefully designed CNN networks trained on synthetic texture images generalize to real-life image data, motivating further pursuit of the proposed approach. To further demonstrate this generalization capability, CNN-based texture directionality detection can be more extensively and, when possible, quantitively demonstrated on real-life image data. This will involve the acquisition of labeled image data targeting texture directionality.

## 5. Summary

The following main achievements were accomplished as part of this study.

We built upon our previous work [29] and created a significantly larger dataset of synthetic texture images with known directionality and perturbation levels (i.e., additive Gaussian noise or averaging kernel-based blurring), feasible for the training and testing of artificial intelligence or other computational tools targeting automated texture directionality detection.

We designed twelve CNN architectures with varying properties. Using the above-mentioned synthetic texture images, we carried out extensive training, validation and testing assays with seven different activation functions. The analysis of the resulting data led to the identification of the best performing CNN network for texture directionality detection, SN1, to be used in combination with the ELU activation function. The network, SN1-ELU, consists of a single convolutional layer of size 17 × 17 × 180 with ELU activation function, global max pooling, and an output layer of size 180 with SM activation function. The network is general, since by design it accommodates images of varying size, even conveniently small.

We subsequently compared the performance of the SN1-ELU network with three state-of-the-art techniques for texture directionality detection, whose implementation is available. Two of techniques are based on LGO and Fourier, and they are implemented as part of the well-known Fiji/ImageJ software [19]. The third technique, based on iGLCM, was designed and implemented in our earlier work on texture directionality detection [29], whose implementation is available in a Github repository [30]. The comparison was carried out based on a separate set of synthetic texture images, different from the one used for training and validation of the CNNs. Data show that SN1-ELU outperforms LGO and Fourier except for the highest blur level, and it is lower but comparable to iGLCM. Based on our tests, the computational efficiency of the SN1-ELU network is superior to the other methods.

Finally, we demonstrated the performance of SN1-ELU on real-life images from the Brodatz repository and from biomedical image repositories. The qualitative assessment of the data clearly shows that our CNN-based model for texture directionality detection, which was trained on synthetic images, nicely generalizes to real-life images.

As pointed out, texture directionality detection is a field with a wealth of applications and with room for further investigation. The novel data and methodologies presented in this paper show the potential of CNN-based approaches. Hence, this work represents a basis for the pursuit and improvement of CNN-based texture directionality detection.

## 6. Disclaimer

Commercial products are identified in this document in order to specify the experimental procedure adequately. Such identification is not intended to imply recommendation or endorsement by the National Institute of Standards and Technology, nor is it intended to imply that the products identified are necessarily the best available for the purpose.

## Figures and Tables

**Table 1:** The four shallow CNN architectures.

#	Layer Type	SN1	SN2	SN3	SN4
1	input (min size x, min size y, channels)	17, 17, 1	17, 17, 1	13, 13, 1	13, 13, 1
2	convolution (size x, size y, count)	17, 17, 180	17, 17, 90	13, 13, 180	13, 13, 90
3	global max pool				
4	dropout	0.25	0.25	0.25	0.25
5	output (count)	180	180	180	180
	Total weights/parameters count	84,780	42,480	63,180	31,680

**Table 2:** The first four deep CNN architectures.

#	Layer Type	DN1	DN2	DN3	DN4
1	input (min size x, min size y, channels)	36, 36, 1	36, 36, 1	36, 36, 1	36, 36, 1
2	convolution (size x, size y, count)	17, 17, 16	17, 17, 16	17, 17, 90	17, 17, 90
3	max pool (size x, size y)	2, 2	2, 2	2, 2	2, 2
4	dropout	0.25	0.25	0.25	0.25
5	convolution (size x, size y, count)	5, 5, 16	5, 5, 32	5, 5, 16	5, 5, 32
6	max pool	2, 2	2, 2	2, 2	2, 2
7	dropout	0.25	0.25	0.25	0.25
8	convolution (size x, size y, count)	3, 3, 90	3, 3, 90	3, 3, 90	3, 3, 90
9	global max pool				
10	dropout	0.25	0.25	0.25	0.25
11	dense (count)	90	90	90	90
12	dropout	0.5	0.5	0.5	0.5
13	output (count)	180	180	180	180
	Total weights/parameters count	48,676	68,052	99,736	148,712

**Table 3:** The second four deep CNN architectures.

#	Layer Type	DN5	DN6	DN7	DN8
1	input (min size x, min size y, channels)	26, 26, 1	26, 26, 1	26, 26, 1	26, 26, 1
2	convolution (size x, size y, count)	7, 7, 16	7, 7, 16	7, 7, 90	7, 7, 90
3	max pool (size x, size y)	2, 2	2, 2	2, 2	2, 2
4	dropout	0.25	0.25	0.25	0.25
5	convolution (size x, size y, count)	5, 5, 16	5, 5, 32	5, 5, 16	5, 5, 32
6	max pool	2, 2	2, 2	2, 2	2, 2
7	dropout	0.25	0.25	0.25	0.25
8	convolution (size x, size y, count)	3, 3, 90	3, 3, 90	3, 3, 90	3, 3, 90
9	global max pool				
10	dropout	0.25	0.25	0.25	0.25
11	dense (count)	90	90	90	90
12	dropout	0.5	0.5	0.5	0.5
13	output (count)	180	180	180	180
	Total weights/parameters count	44,836	56,562	78,136	127,112

**Table 4:** Performance comparison (RMSE (°) and speed (tiles/s)) between CNN, iGLCM Fourier and LGO-based directionality detection (same testing set).

	**SN1-ELU**	**iGLCM**	**Fourier**	**LGO**
**Gaussian Noise Std Value**	**RMSE (°)**	**RMSE (°)**	**RMSE (°)**	**RMSE (°)**
none	0.00	0.00	1.58	2.03
2000	0.01	0.00	1.70	2.11
4000	0.06	0.07	2.11	2.11
6000	0.23	0.16	2.93	2.43
8000	0.48	0.31	5.64	2.69
	**SN1-ELU**	**iGLCM**	**Fourier**	**LGO**
**Blur Kernel Size**	**RMSE (°)**	**RMSE (°)**	**RMSE (°)**	**RMSE (°)**
none	0.00	0.00	1.58	2.03
3 × 3	0.01	0.00	2.42	2.61
5 × 5	0.07	0.00	3.16	1.27
7 × 7	2.79	2.86	4.41	3.41
9 × 9	5.81	6.00	5.29	3.91
	**SN1-ELU**	**iGLCM**	**Fourier**	**LGO**
**# Tiles Tested**	**(tiles/s)**	**(tiles/s)**	**(tiles/s)**	**(tiles/s)**
178,605	6613.3	34.4	902.2	902.1

## Data Availability

The training, testing and validation data used in this work, consisting of synthetic textures, can be obtained following the procedure described in Section 2.1. The data amounts to about 12 Gb.

## Supplementary Materials

The following supporting information can be downloaded at: https://www.mdpi.com/article/10.3390/s22020562/s1, Figure S1: Instances of shallow and deep CNN architectures; Figure S2: Activation functions tested in this work; Figure S3: Training and validation loss curves for the three replicas of SN1 ReLU; Figure S4: Training and validation loss curves for the three replicas of SN1 ELU; Figure S5: Training and validation loss curves for the three replicas of SN1 SELU; Figure S6: Training and validation loss curves for the three replicas of SN1 Si; Figure S7: Training and validation loss curves for the three replicas of SN1 SP; Figure S8: Training and validation loss curves for the three replicas of SN1 SS; Figure S9: Training and validation loss curves for the three replicas of TanH; Figure S10: Training and validation loss curves for the SN1; Figure S11: Training and validation loss curves for the SN2; Figure S12: Training and validation loss curves for the SN3; Figure S13: Training and validation loss curves for the SN4; Figure S14: Training and validation loss curves for the DN1; Figure S15: Training and validation loss curves for the DN2; Figure S16: Training and validation loss curves for the DN3; Figure S17: Training and validation loss curves for the DN4; Figure S18: Training and validation loss curves for the DN5; Figure S19: Training and validation loss curves for the DN6; Figure S20: Training and validation loss curves for the DN7; Figure S21: Training and validation loss curves for the DN8; Figure S22: CNN-based directionality detection on microcopy images of actin-stained fibroblast cells; Figure S23: CNN-based directionality detection on Brodatz textures [52,59,60] and corresponding polar plots; Figure S24: Directionality RMSE vs. Gaussian noise standard deviation for all shallow and deep CNNs; Figure S25: Directionality RMSE vs. blur kernel size for all shallow and deep CNNs; Figure S26: Filters from the only convolutional layer of the best shallow CNN, SN1-ELU for the three training replicates; Figure S27: Filters from the convolutional layers of the best deep CNN, DN2-TanH for the three training replicates; Figure S28: Instances of synthetic and real-life textures with complex signal; Figure S29: Instances of convolutions of synthetic textures with kernels from the first layer of the deep network DN2; Figure S30: Instances of convolutions of synthetic textures with kernels from the first layer of the deep network DN6; Table S1: Performance comparison between the best shallow and deep CNNs; Table S2: Directionality RMSE values vs. Gaussian noise standard deviation and averaging blur for deep networks DN2-TanH and DN6-TanH.
