# Supplementary material for: A Convolutional Neural Networks-Based Approach for Texture Directionality Detection
Source: Sensors (Basel). 2022 Jan 12;22(2):562. doi: 10.3390/s22020562 (PMC8778371; doi:10.3390/s22020562)
Supplement: Supplementary file 1 [file sensors-22-00562-s001.zip › sensors-1477693-supplementary.pdf]

A Convolutional Neural Networks-Based Approach for Texture Directionality Detection - Supplementary Materials

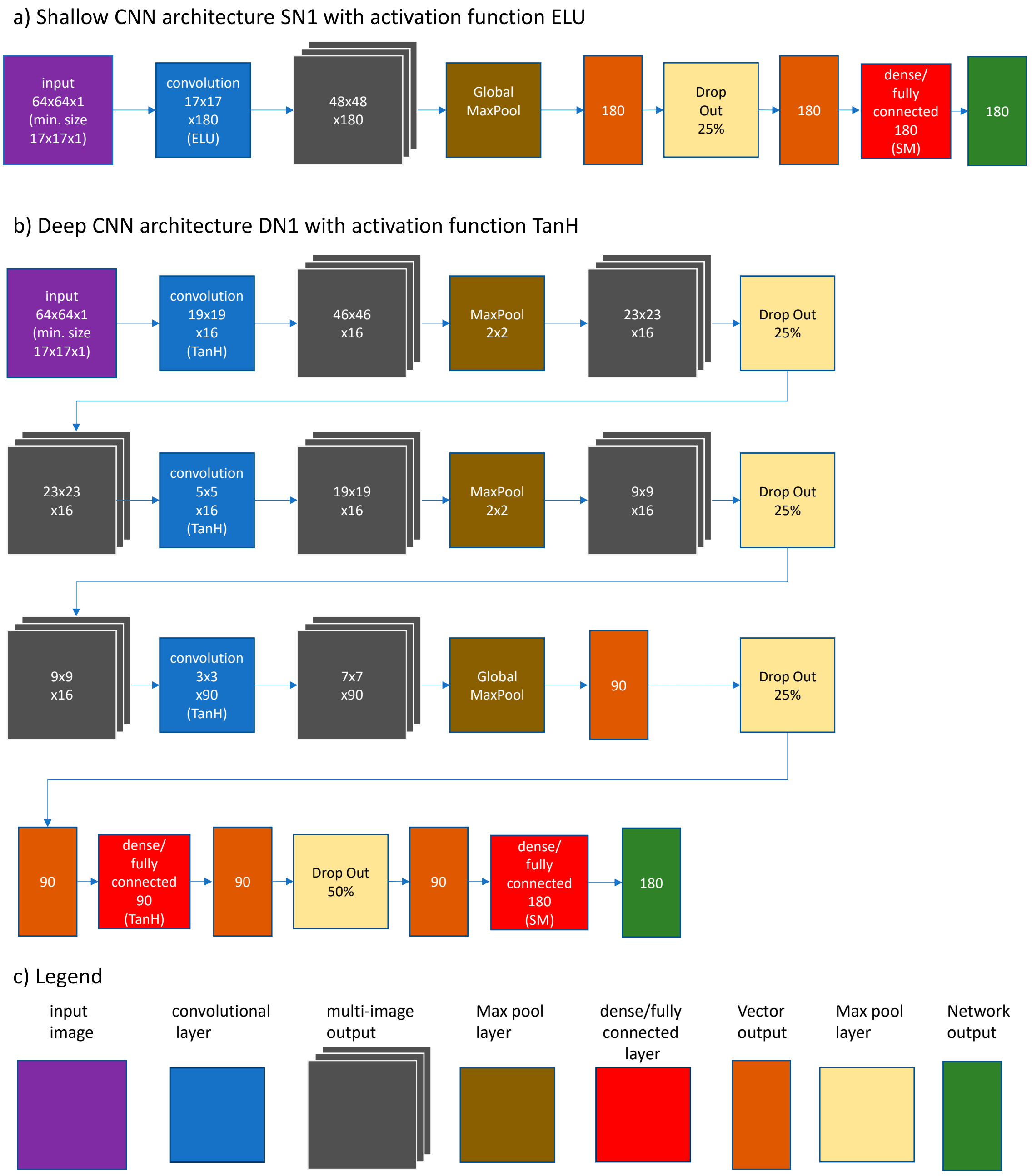

Figure S1. *Instances of shallow (a) and deep (b) CNN architectures and legend for the diagram blocks (c). In the "convolutional layer" and "dense/fully connected layer" blocks an activation function is reported in parenthesis. The quantities in the "input image" and "multi-image output" blocks will change depending on the size of the input image. The specific quantities shown in the figure represent the size of the images processed in the paper. In the "input image" block the minimum allowed image size is reported in parenthesis.*

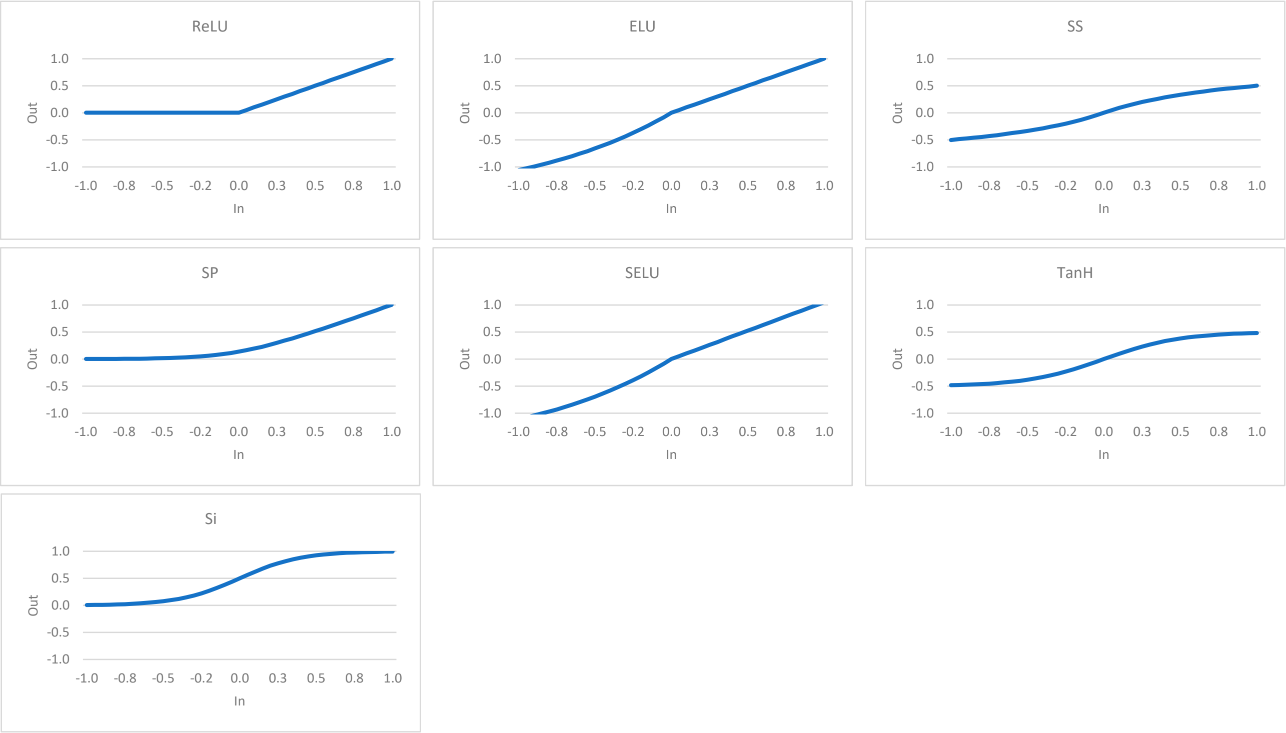

Figure S2. *Activation functions tested in this work; graphs obtained from equations in [56].*

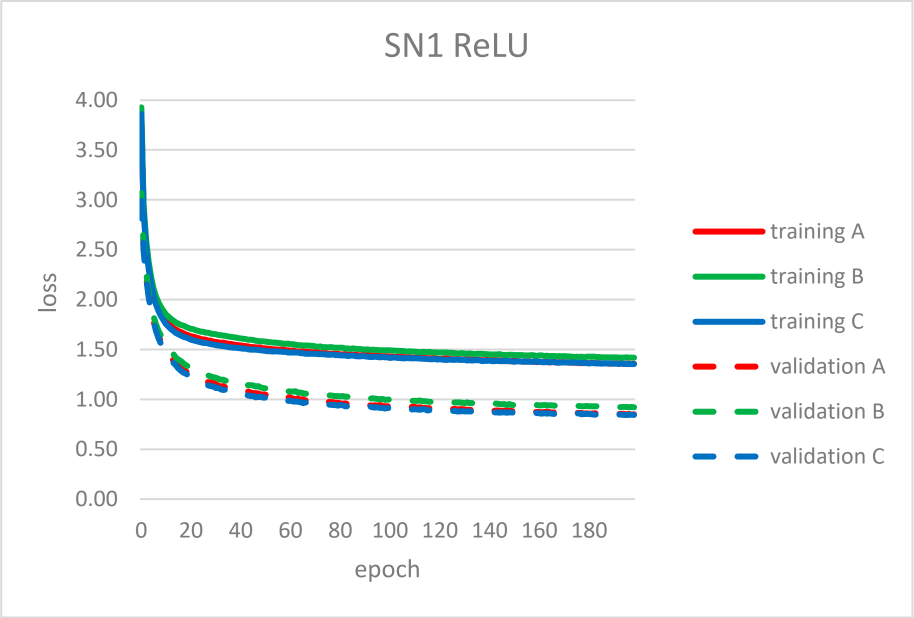

Figure S3 *Training and validation loss curves for the three replicas of SN1 ReLU.*

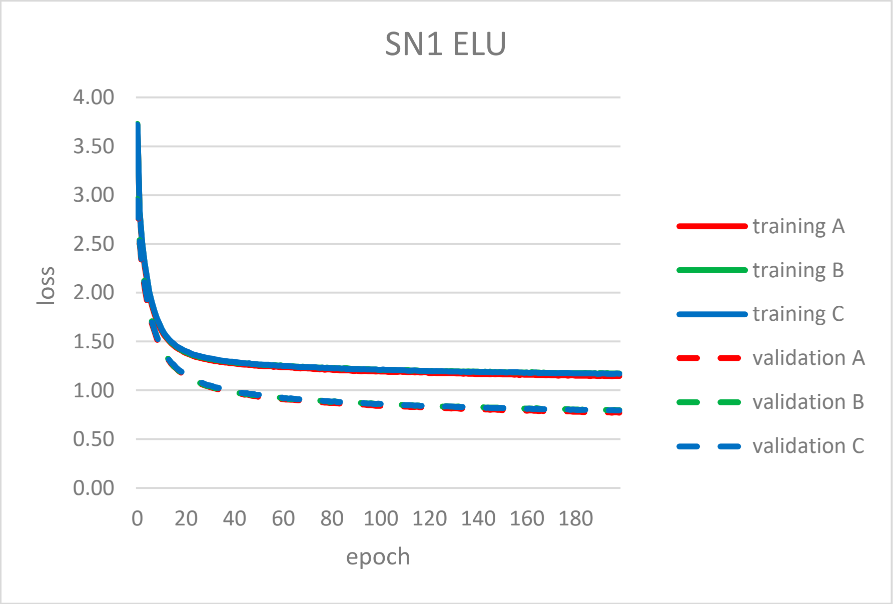

Figure S4. *Training and validation loss curves for the three replicas of SN1 ELU.*

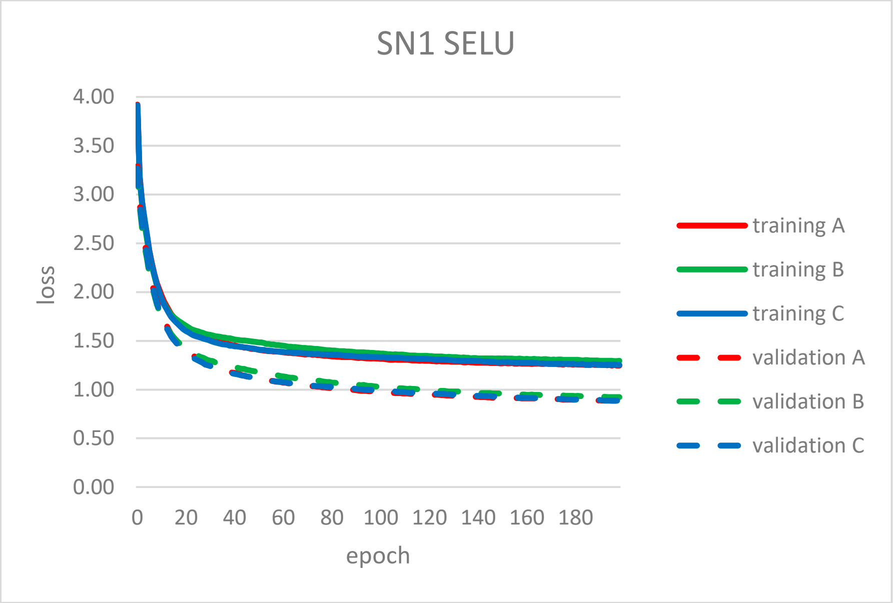

Figure S5. *Training and validation loss curves for the three replicas of SN1 SELU.*

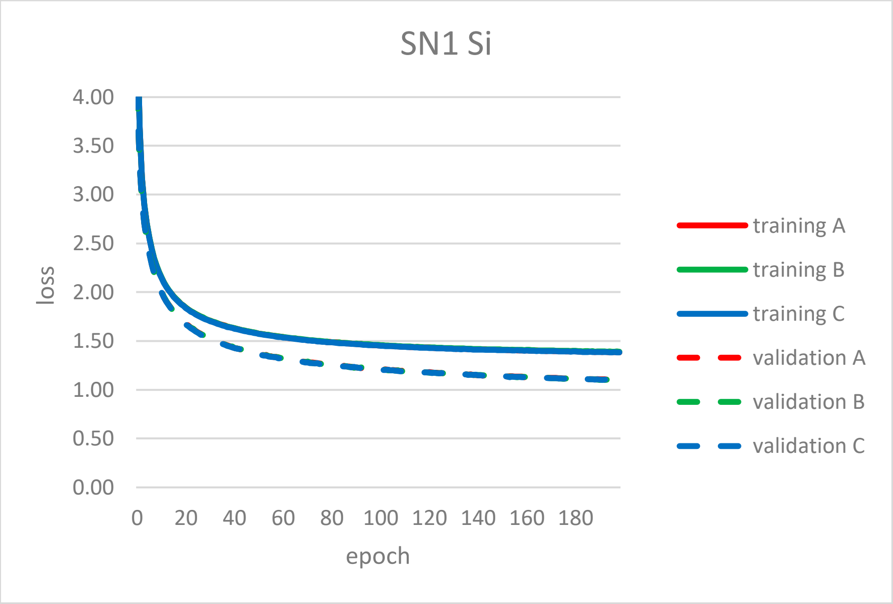

Figure S6. *Training and validation loss curves for the three replicas of SN1 Si.*

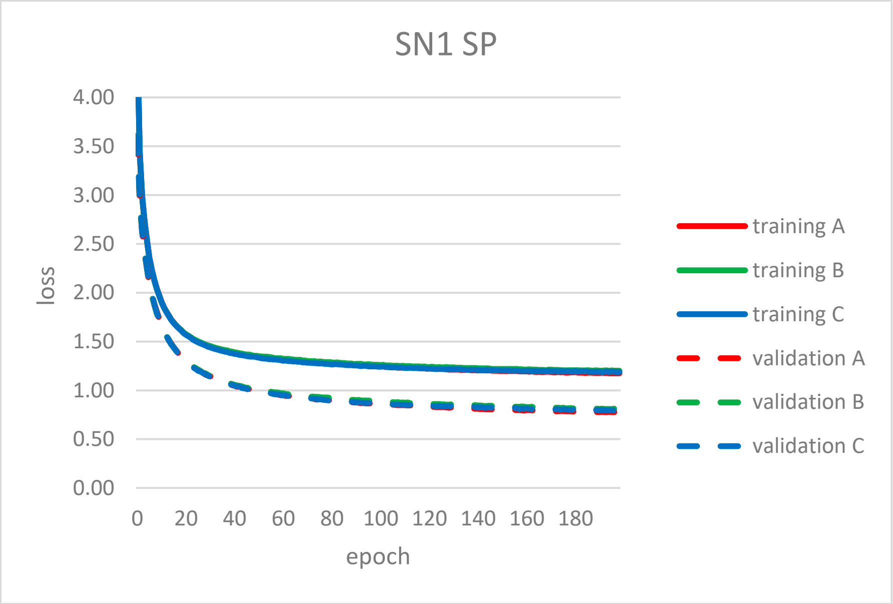

Figure S7. *Training and validation loss curves for the three replicas of SN1 SP.*

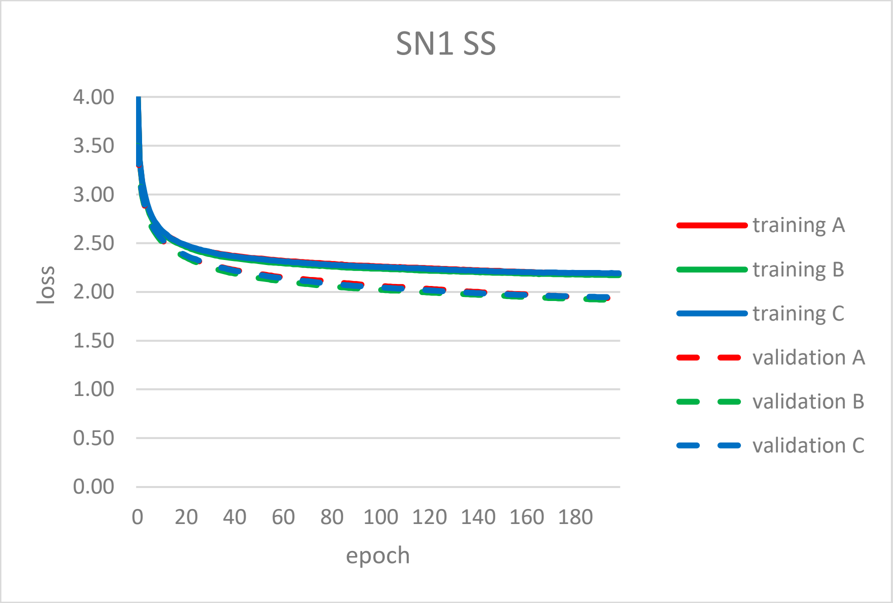

Figure S8. *Training and validation loss curves for the three replicas of SN1 SS.*

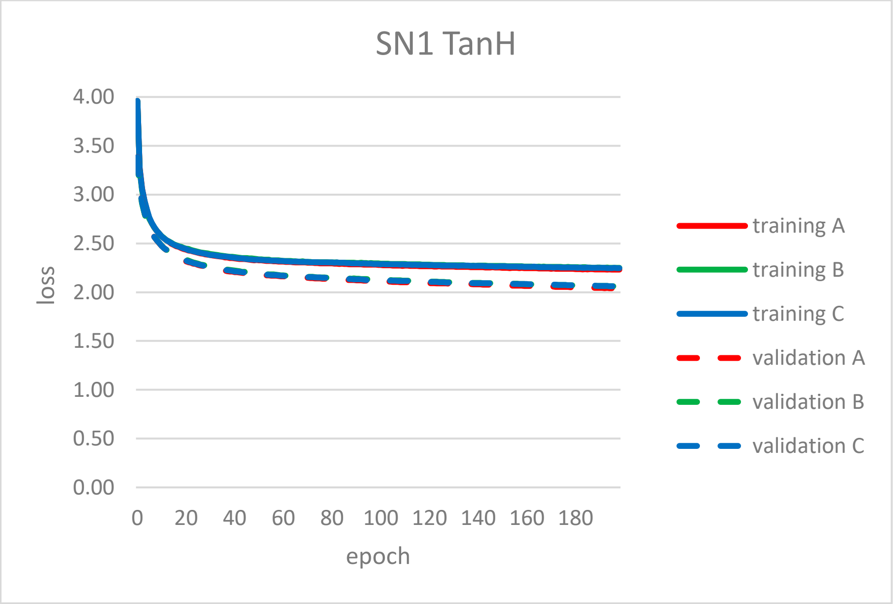

Figure S9. *Training and validation loss curves for the three replicas of TanH.*

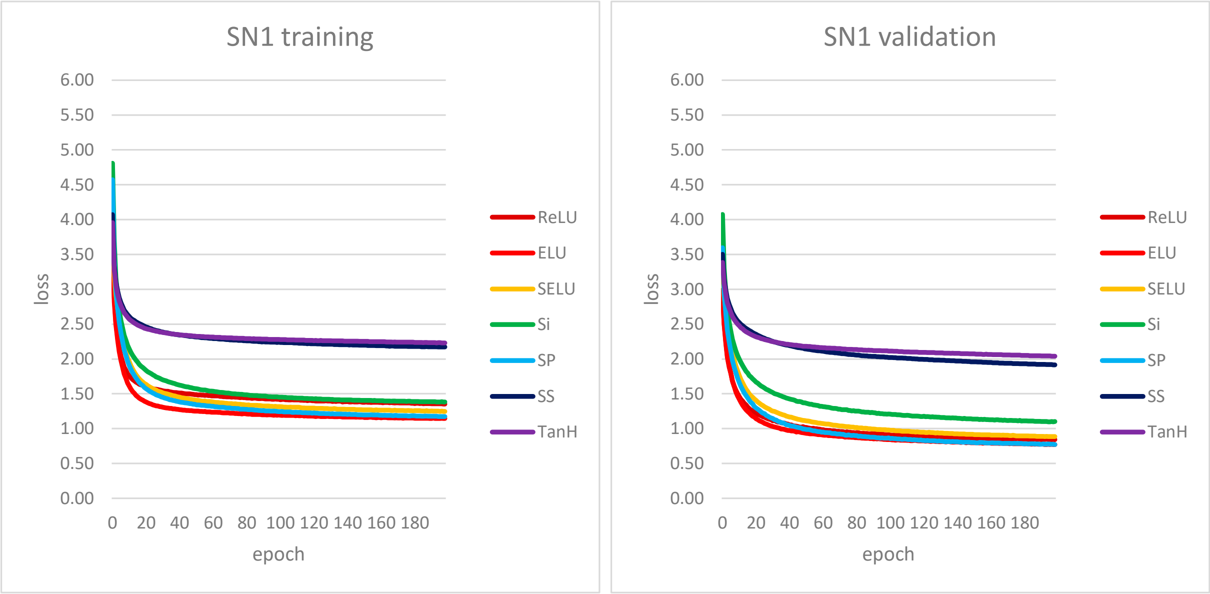

Figure S10. *Training and validation loss curves for the SN1.*

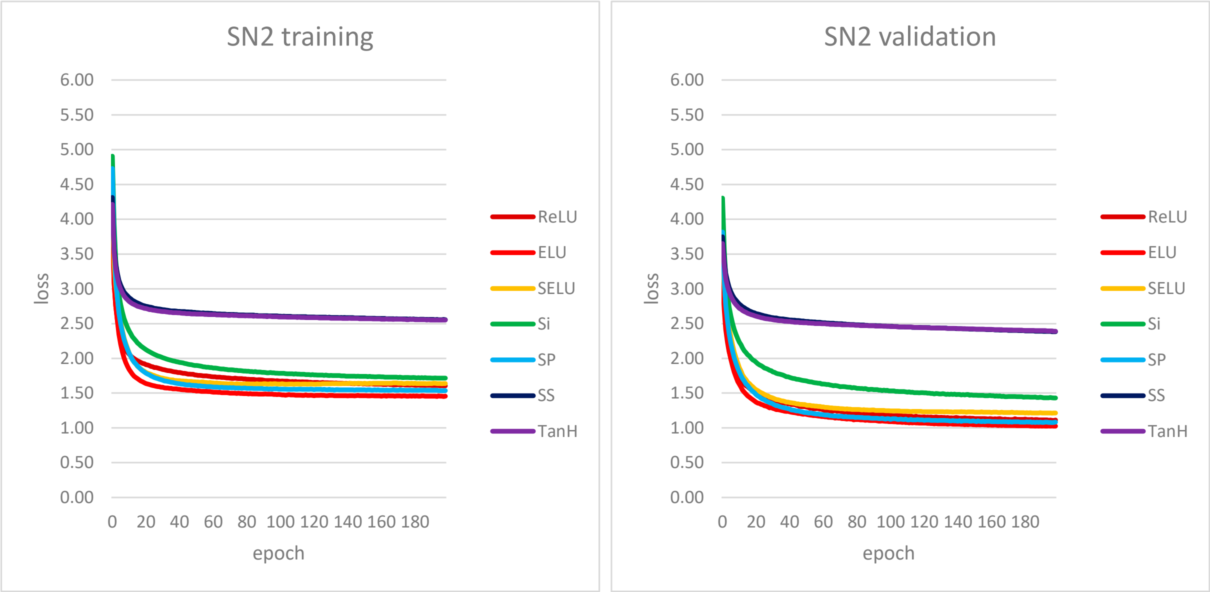

Figure S11. *Training and validation loss curves for the SN2.*

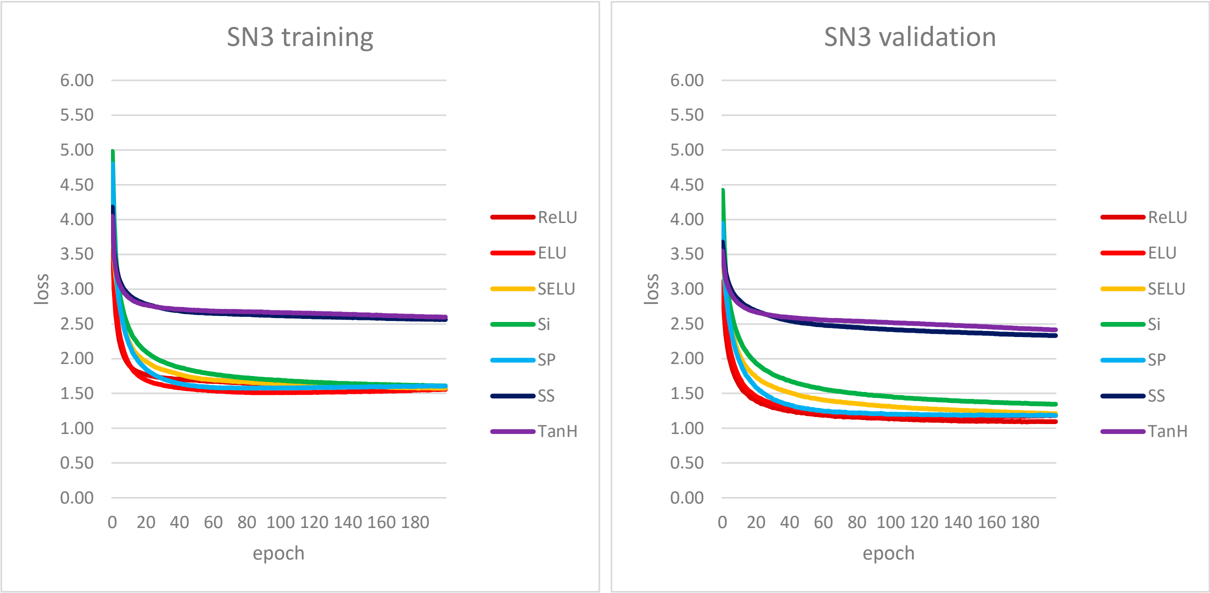

Figure S12. *Training and validation loss curves for the SN3.*

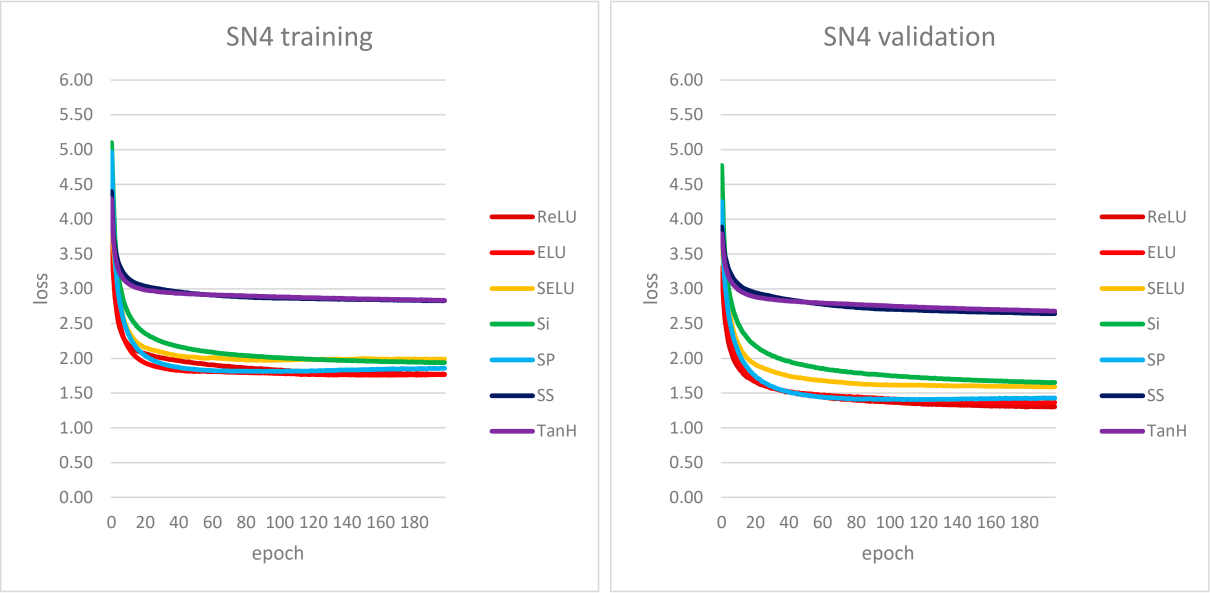

Figure S13. *Training and validation loss curves for the SN4.*

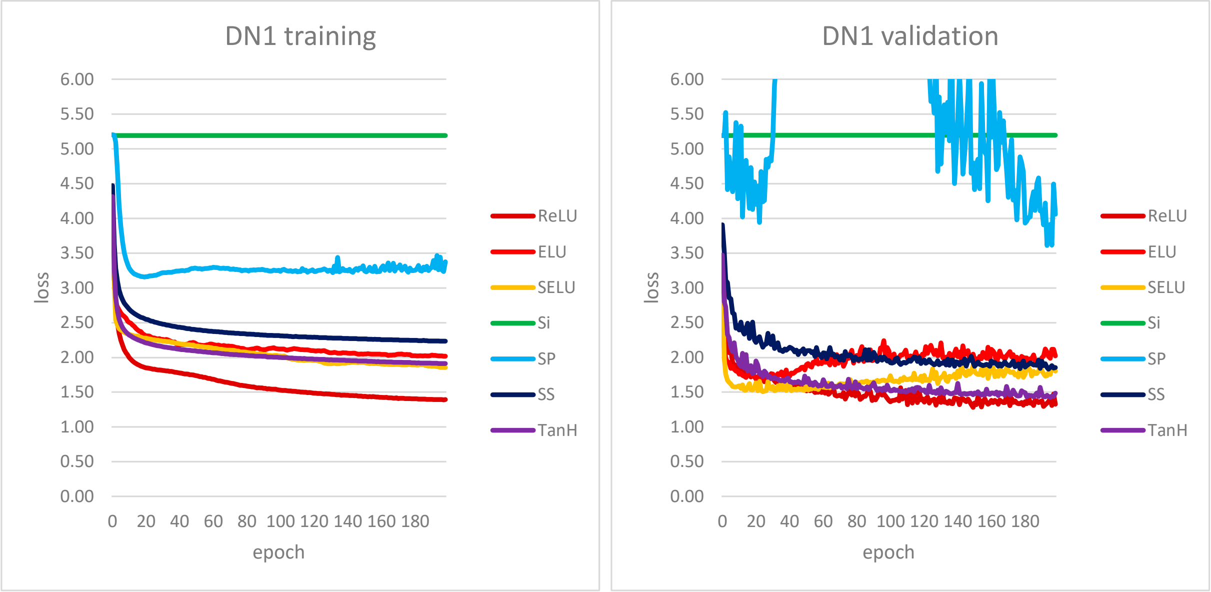

Figure S14. *Training and validation loss curves for the DN1.*

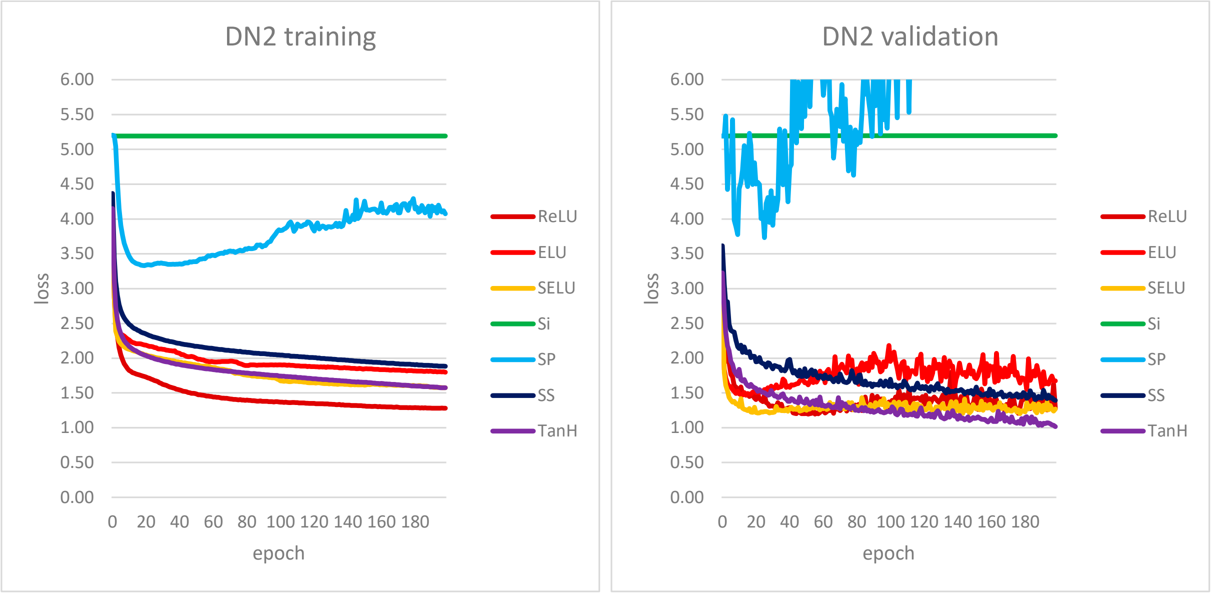

Figure S15. *Training and validation loss curves for the DN2.*

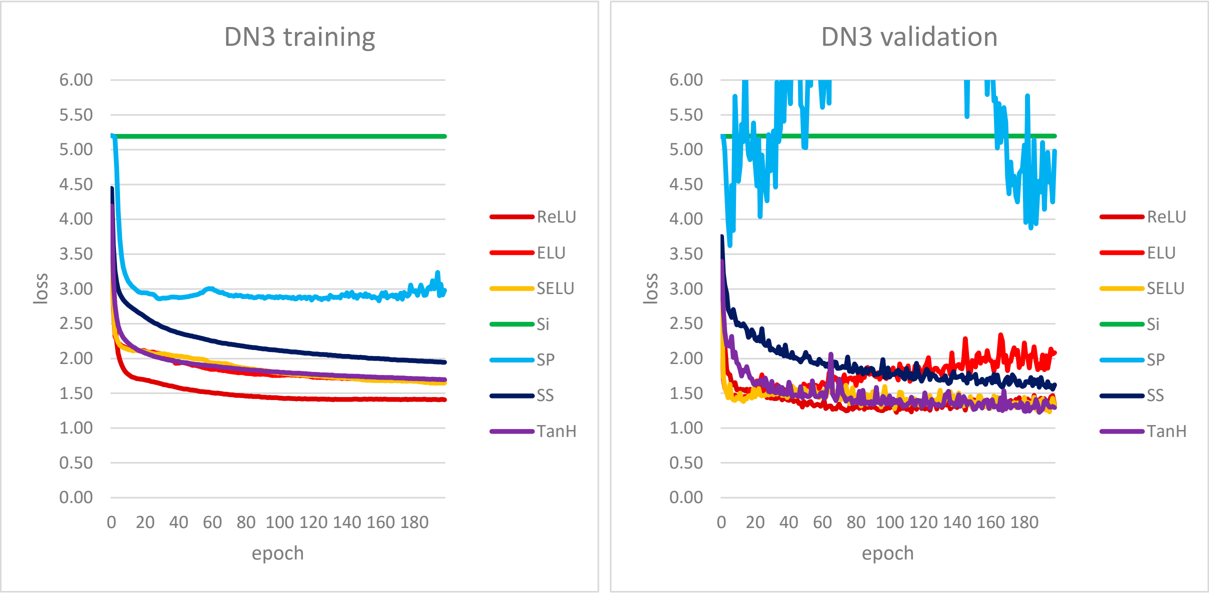

Figure S16. *Training and validation loss curves for the DN3.*

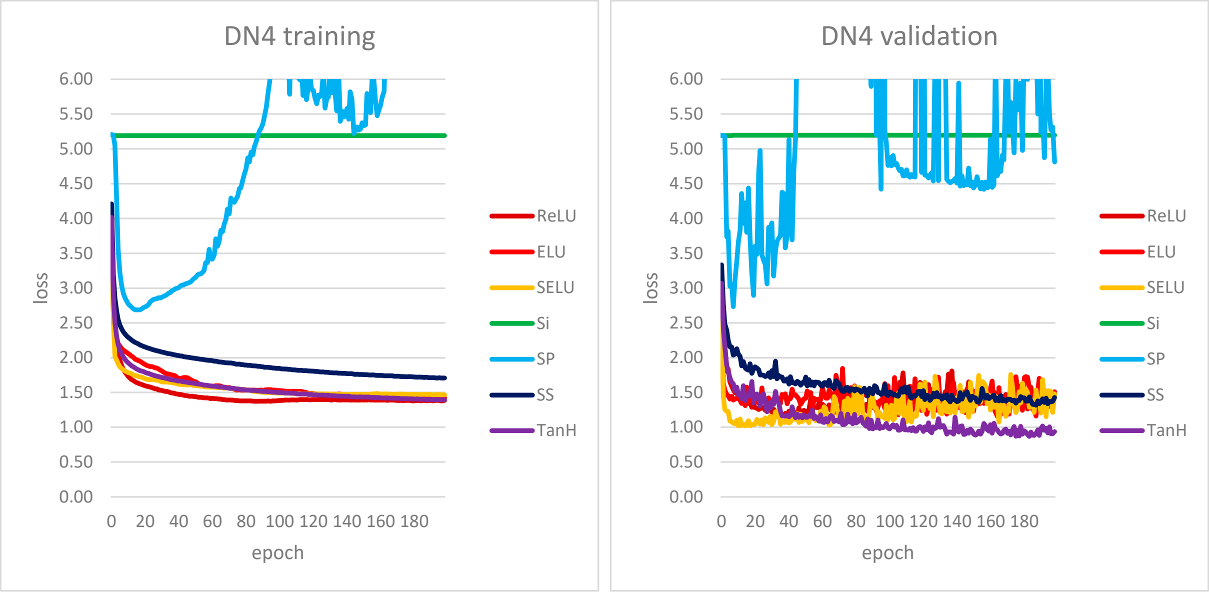

Figure S17. *Training and validation loss curves for the DN4.*

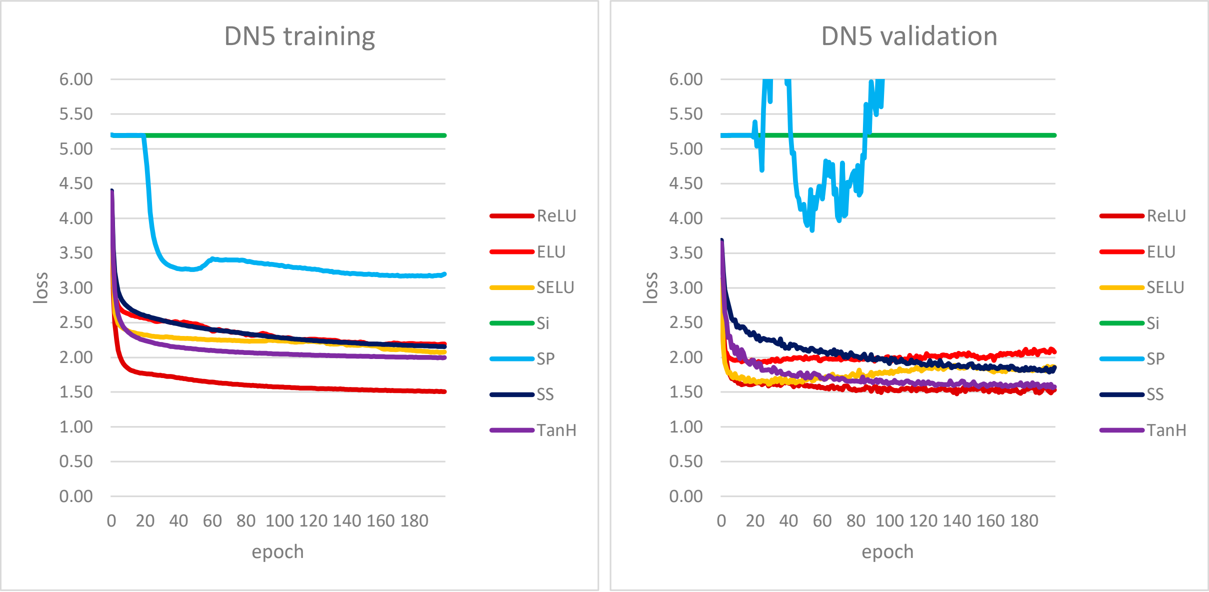

Figure S18. *Training and validation loss curves for the DN5.*

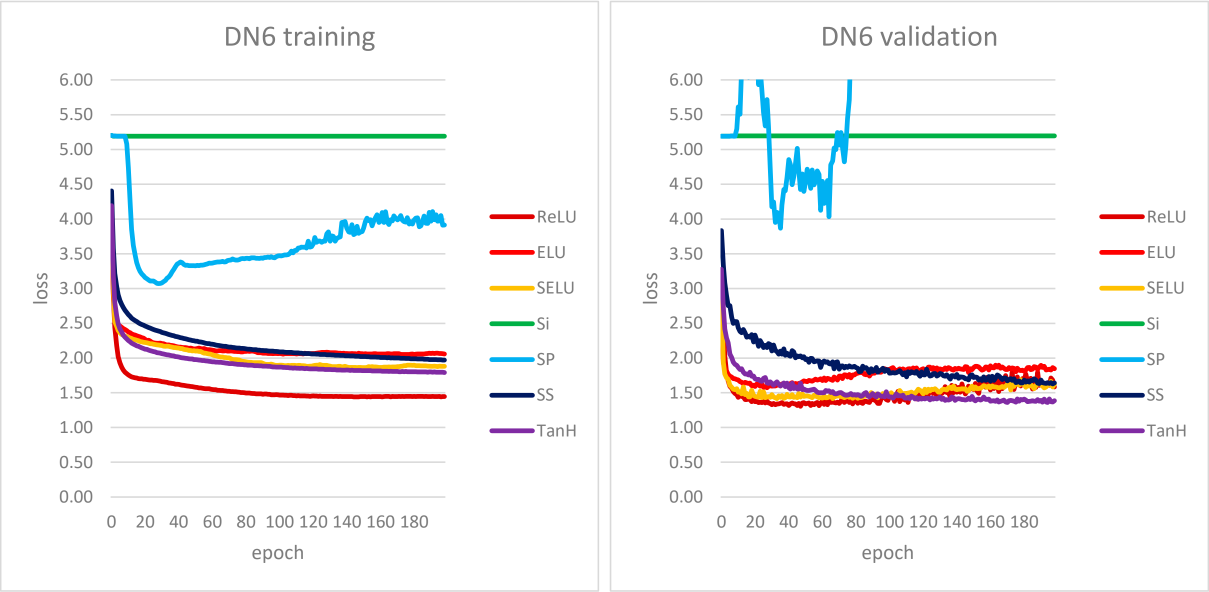

Figure S19. *Training and validation loss curves for the DN6.*

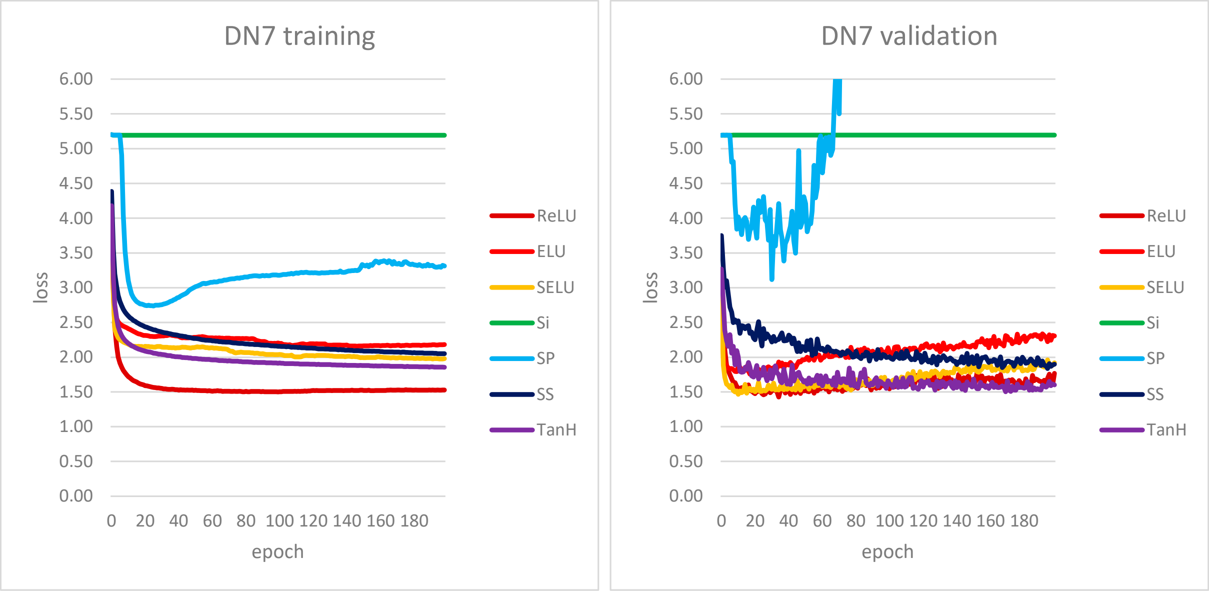

Figure S20. *Training and validation loss curves for the DN7.*

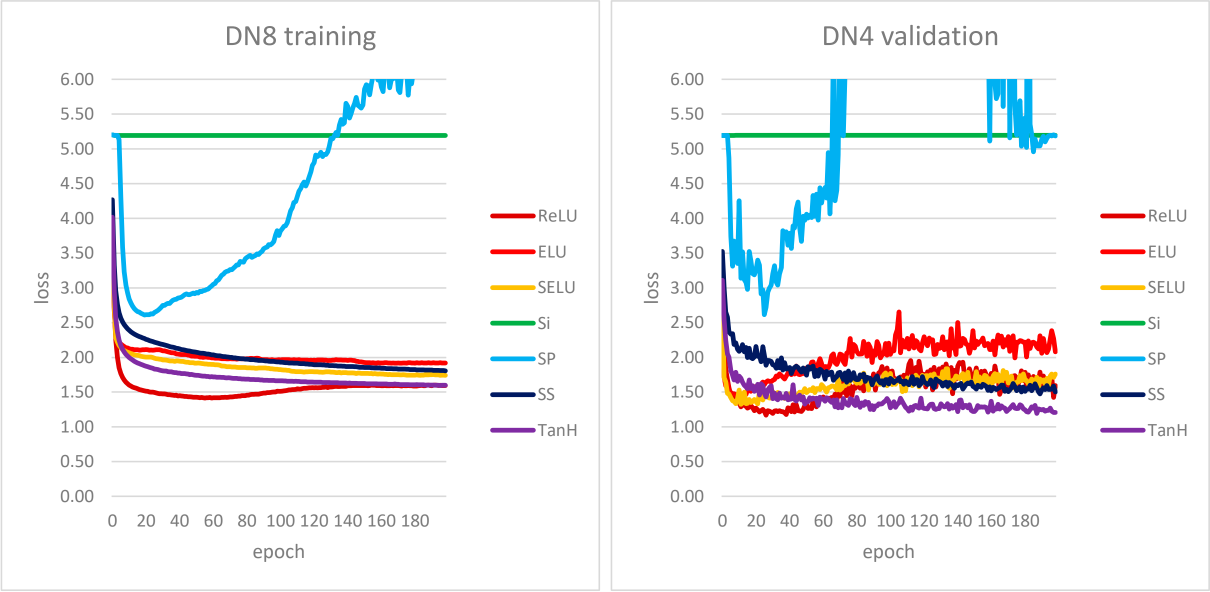

Figure S21. *Training and validation loss curves for the DN8.*

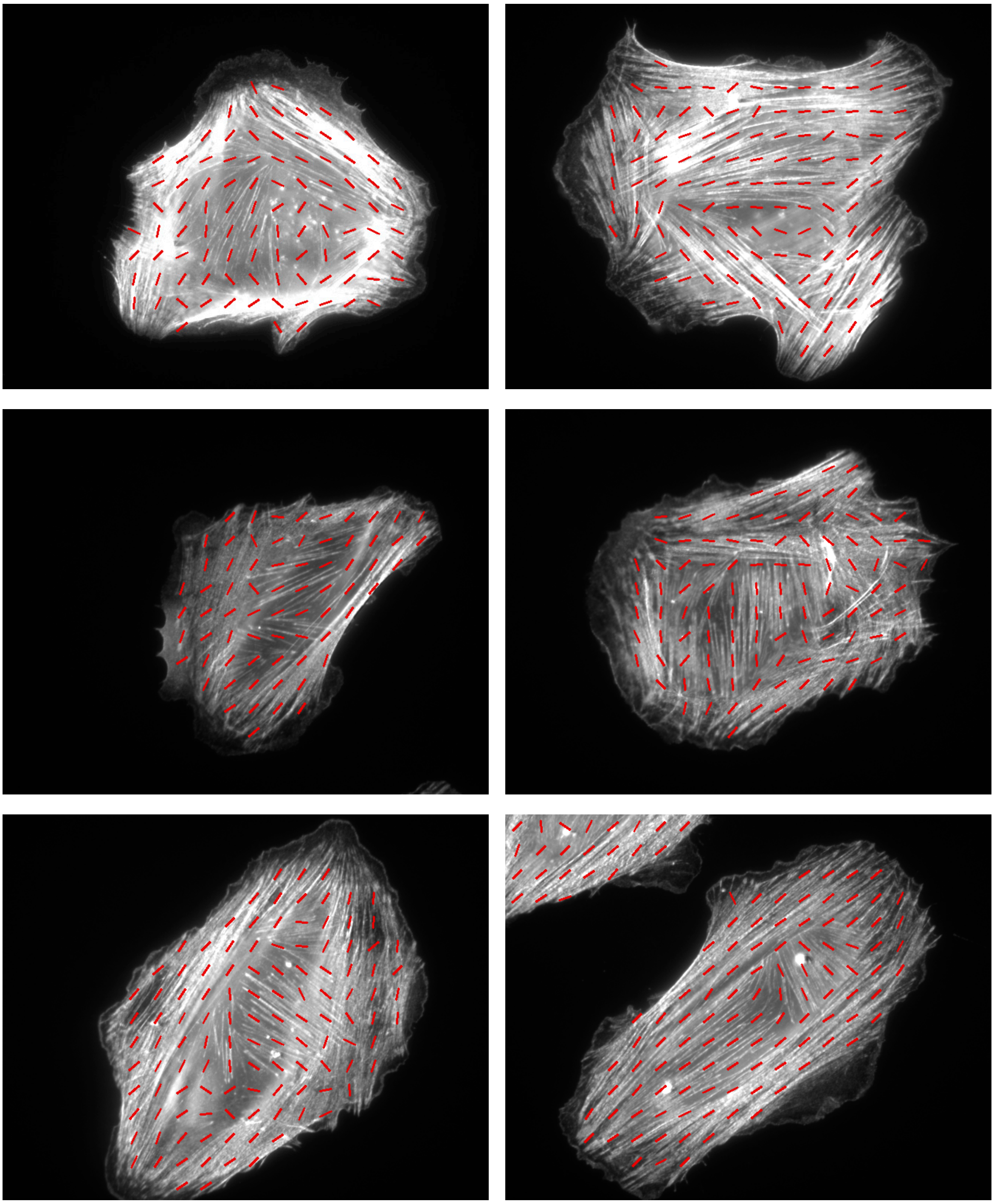

Figure S22. *CNN-based directionality detection on microcopy images of actin-stained fibroblast cells.*

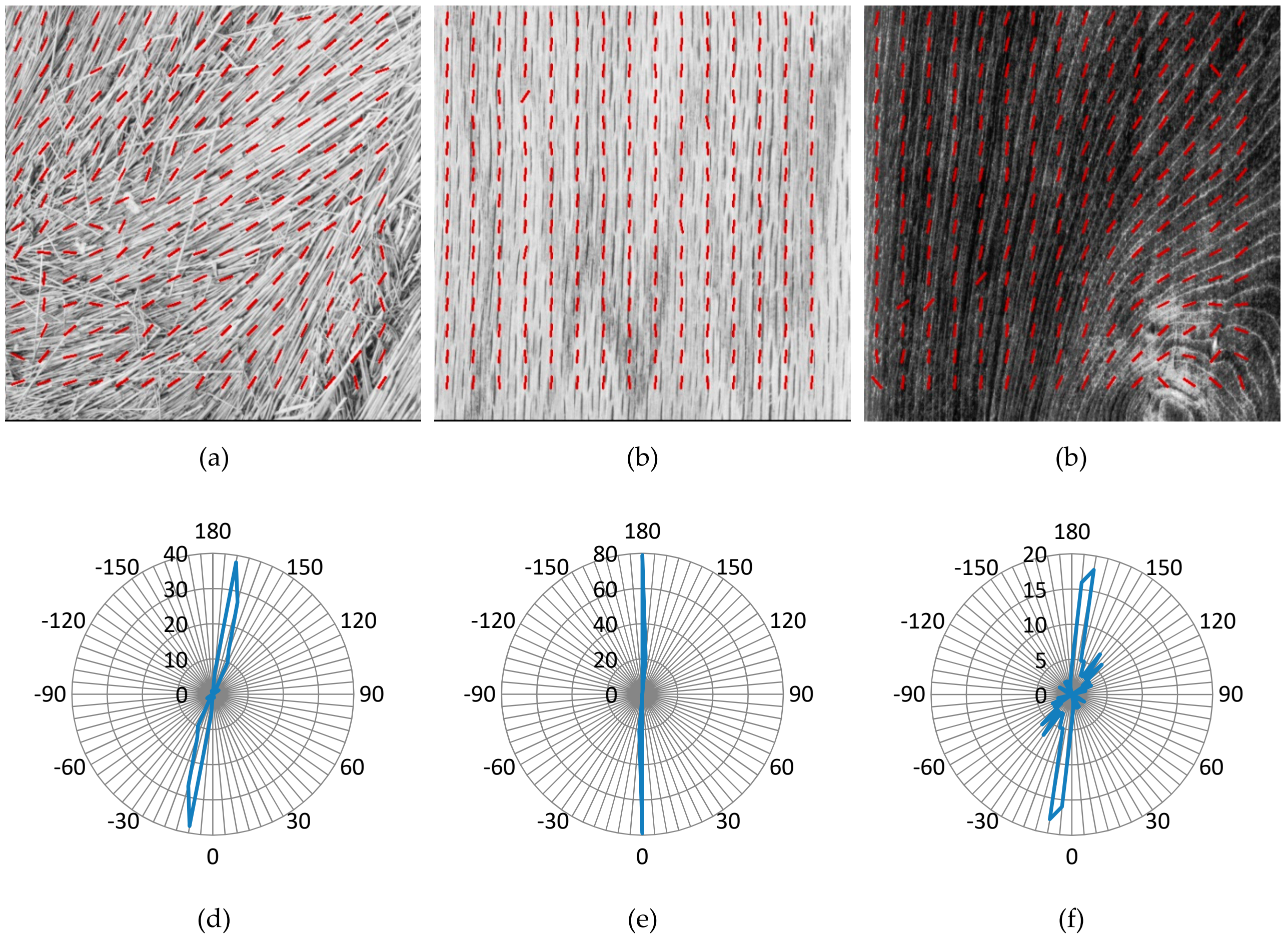

Figure S23. CNN-based directionality detection on: (a), (b), (c) Brodatz textures [52,59,60] and corresponding polar plots (d), (e), (f) representing the direction distribution in the analyzed textures.

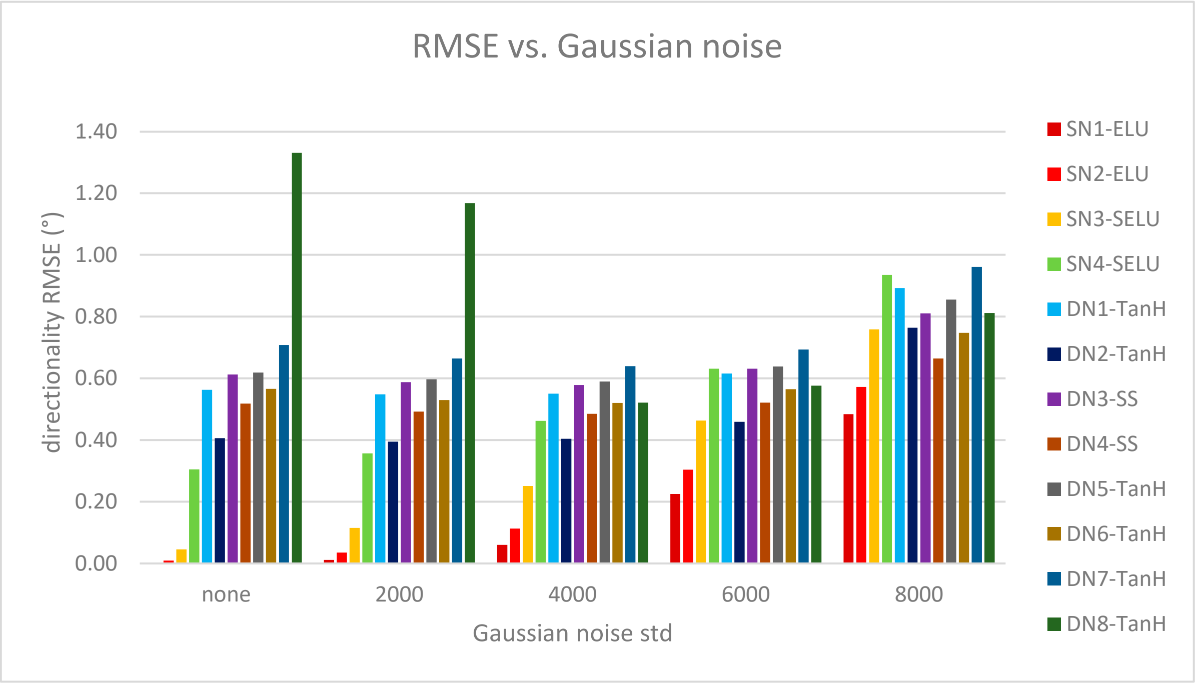

Figure S24. *Directionality RMSE vs. Gaussian noise standard deviation for all shallow and deep CNNs. The RMSE for each CNN is computed with the respective best performing activation function.*

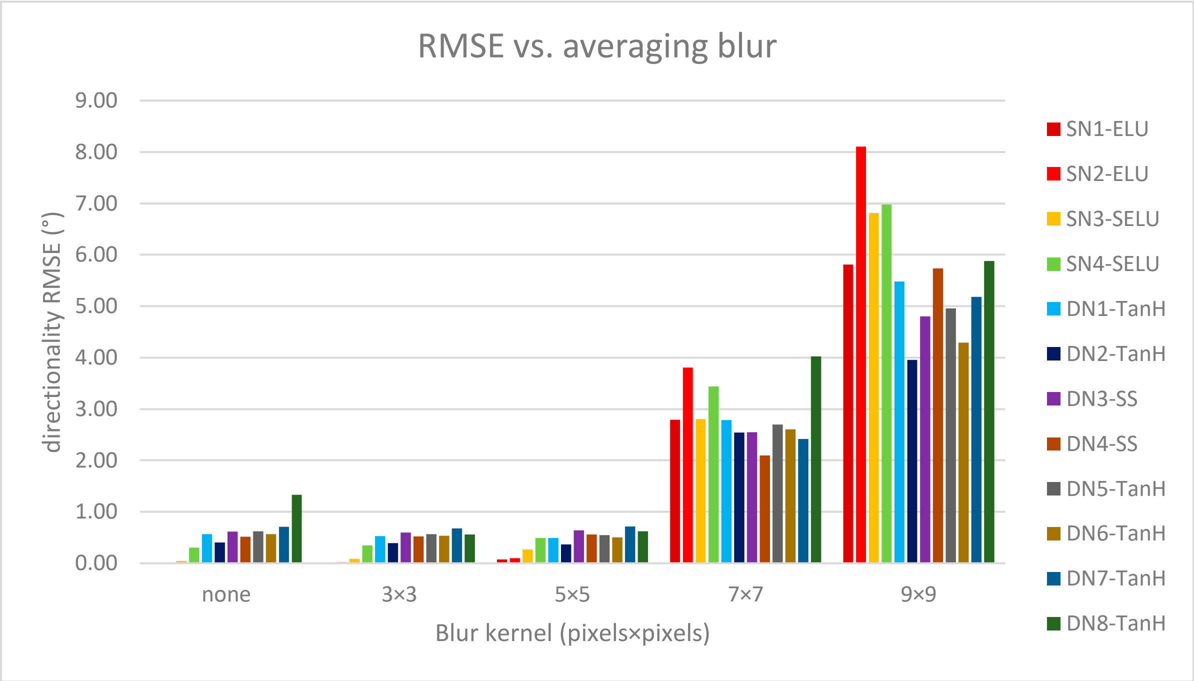

Figure S25. *Directionality RMSE vs. blur kernel size for all shallow and deep CNNs. The RMSE for each CNN is computed with the respective best performing activation function.*

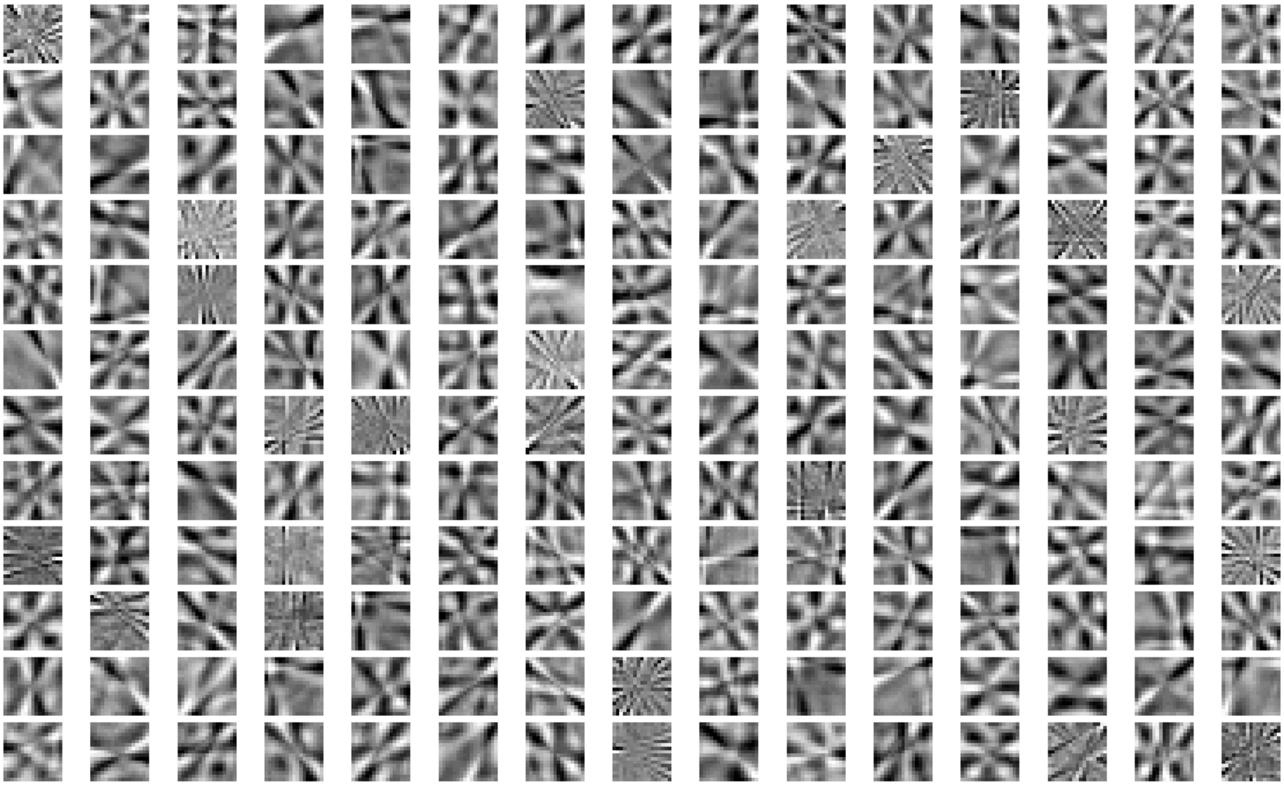

(a)

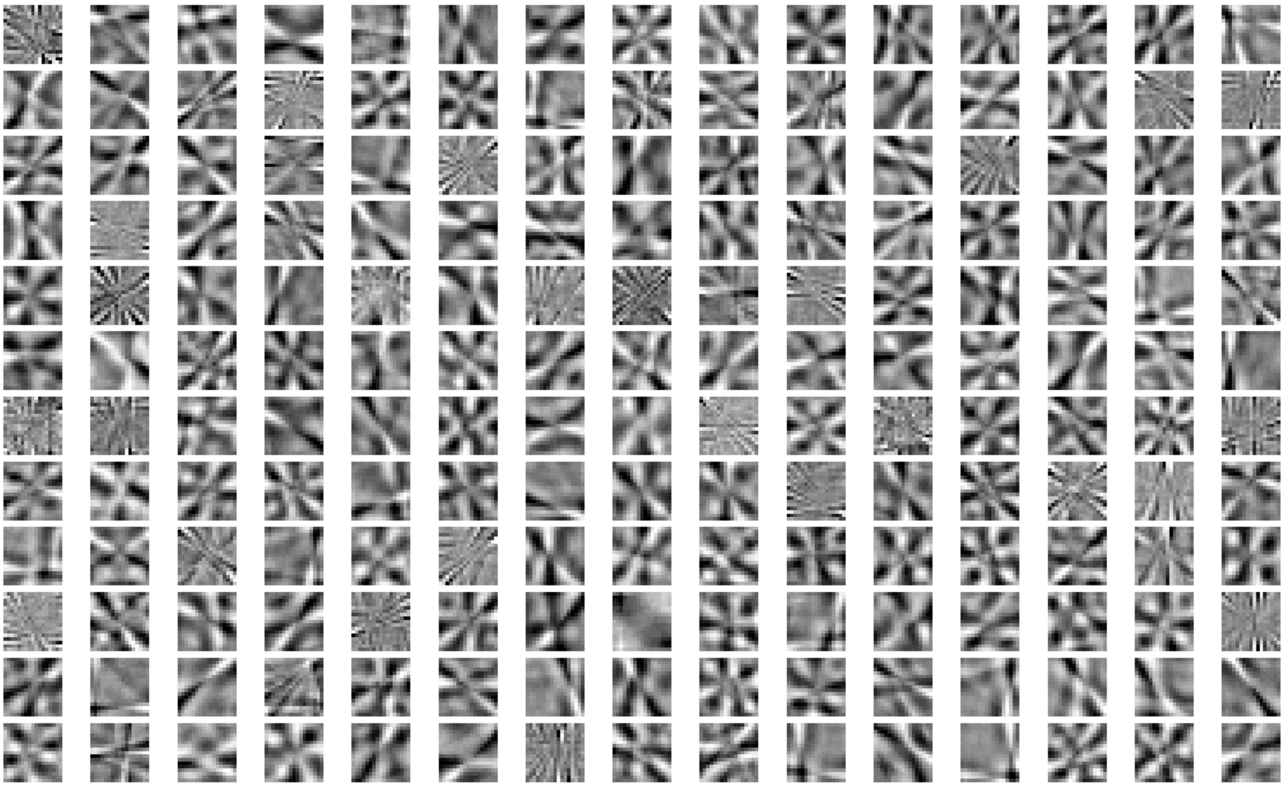

(b)

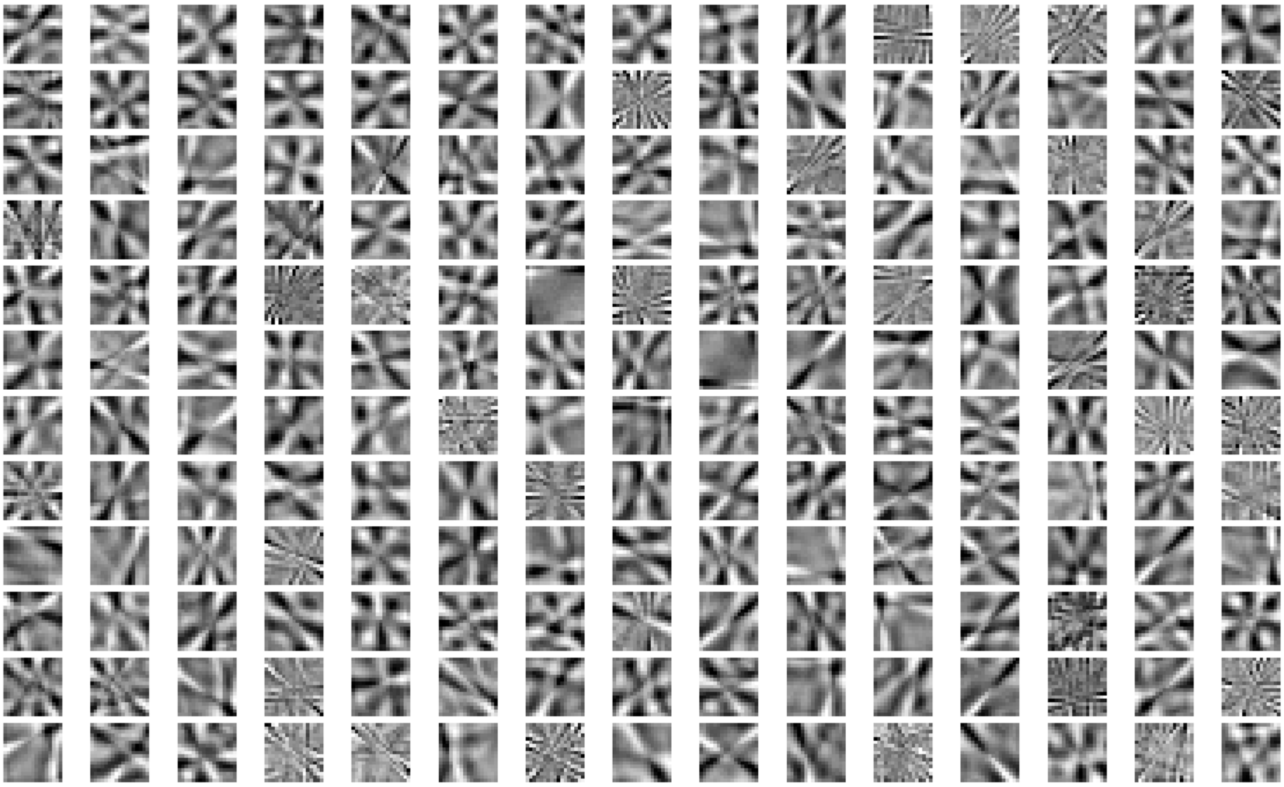

(c)

Figure S26. *Filters from the only convolutional layer of the best shallow CNN, SN1-ELU, for the three training instances (a, b, c, 180 filters each).*

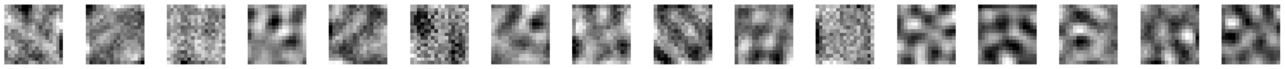

(a) DN2-TanH, first training replicate, first convolutional layer

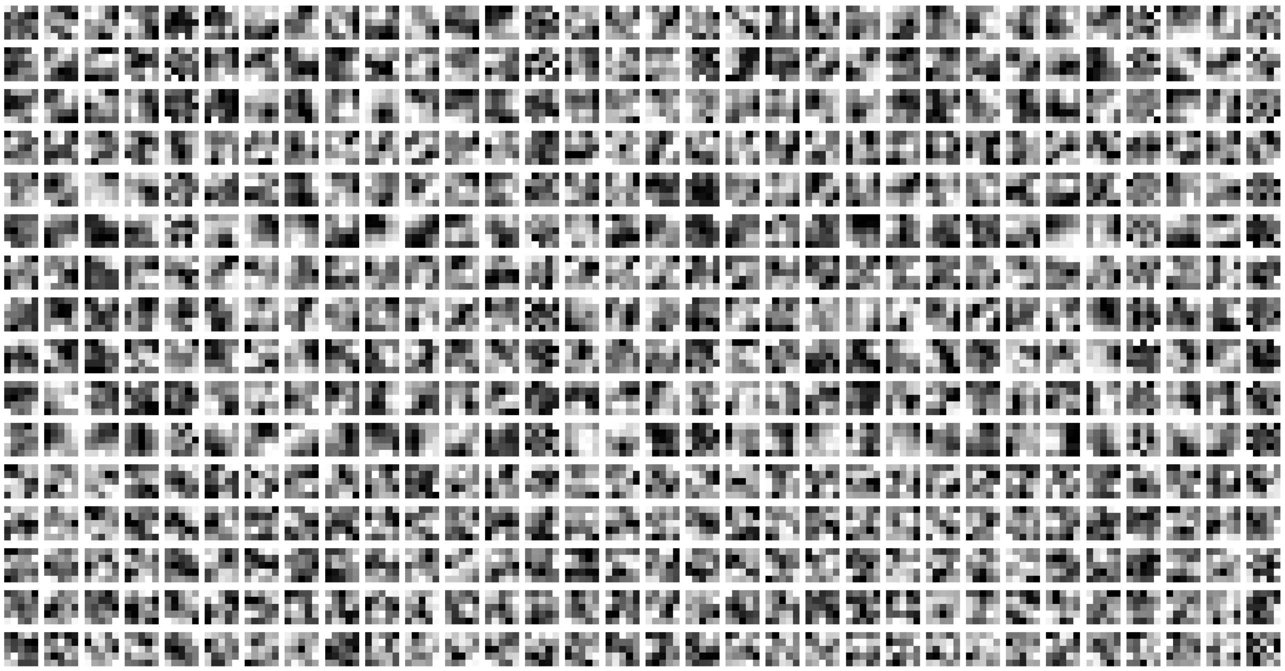

(b) DN2-TanH, first training replicate, second convolutional layer

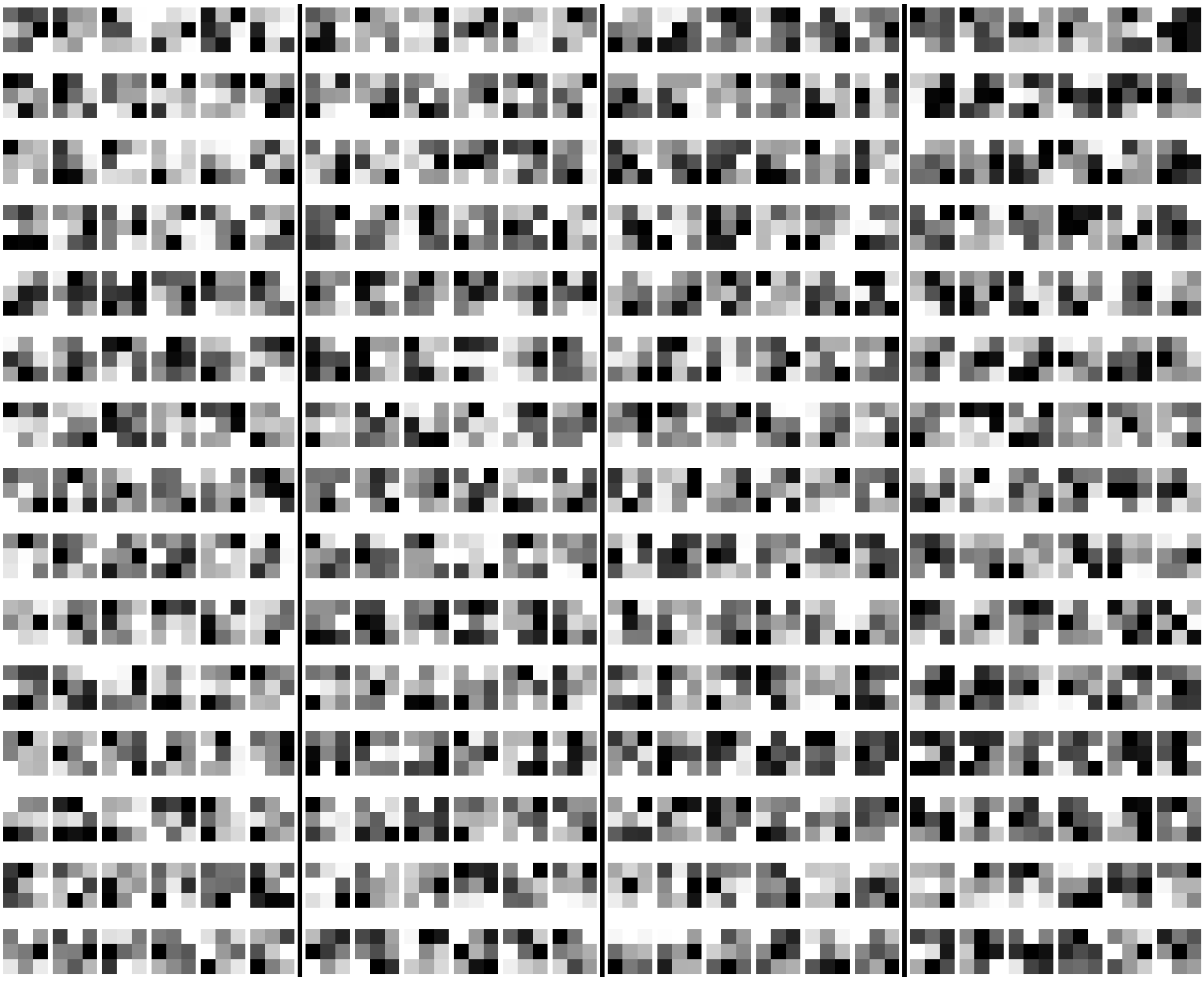

(c) DN2-TanH, first training replicate, third convolutional layer, 4 filter groups out of 32 shown

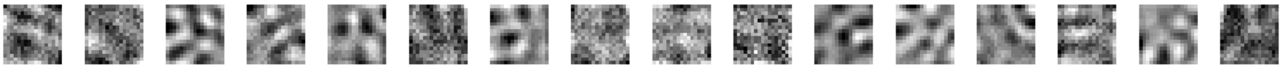

(d) DN2-TanH, second training replicate, first convolutional layer

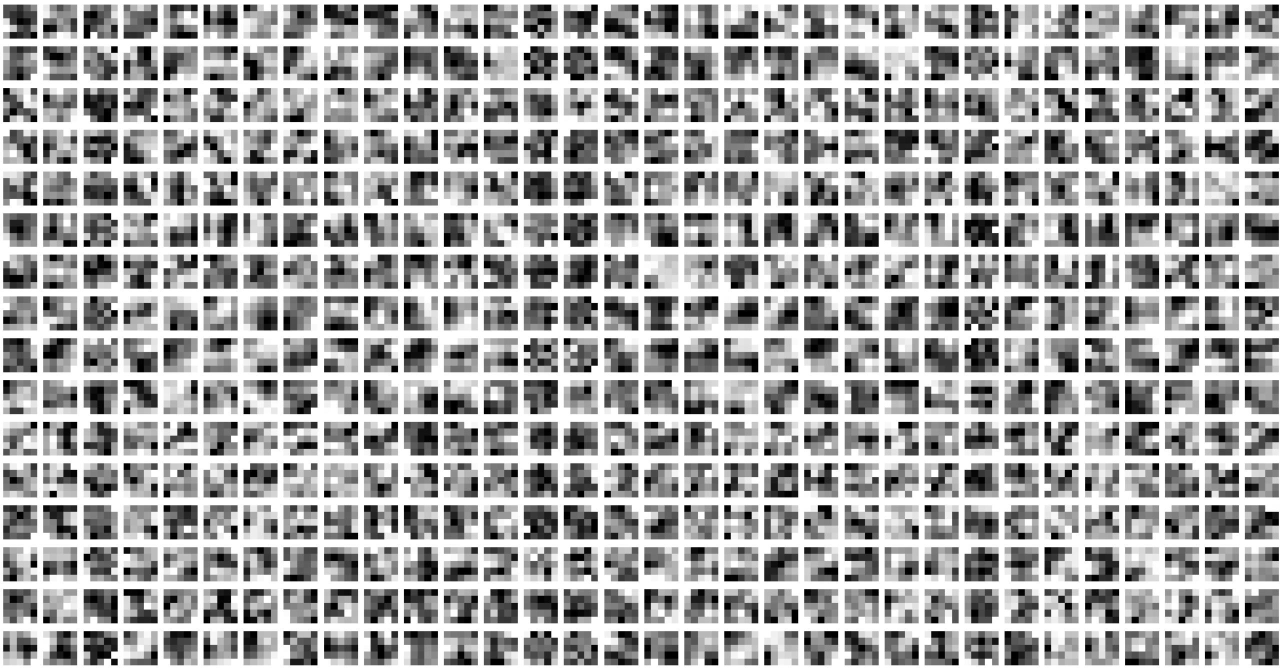

(e) DN2-TanH, second training replicate, second convolutional layer

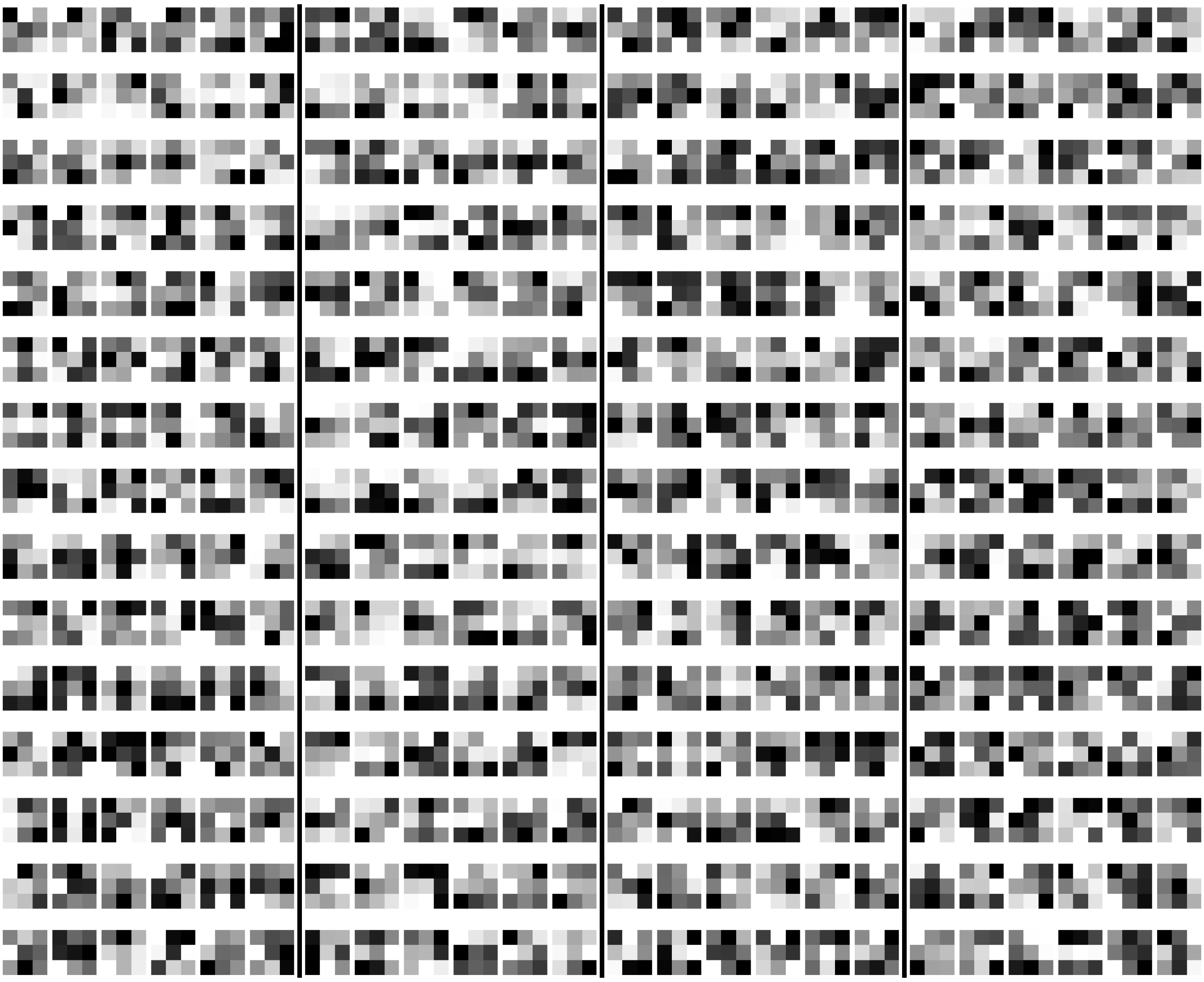

(f) DN2-TanH, second training replicate, third convolutional layer, 4 filter groups out of 32 shown

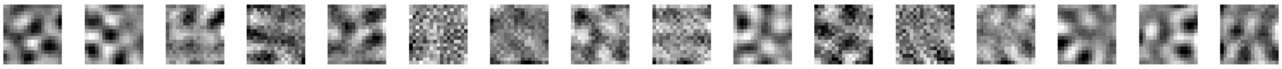

(g) DN2-TanH, third training replicate, first convolutional layer

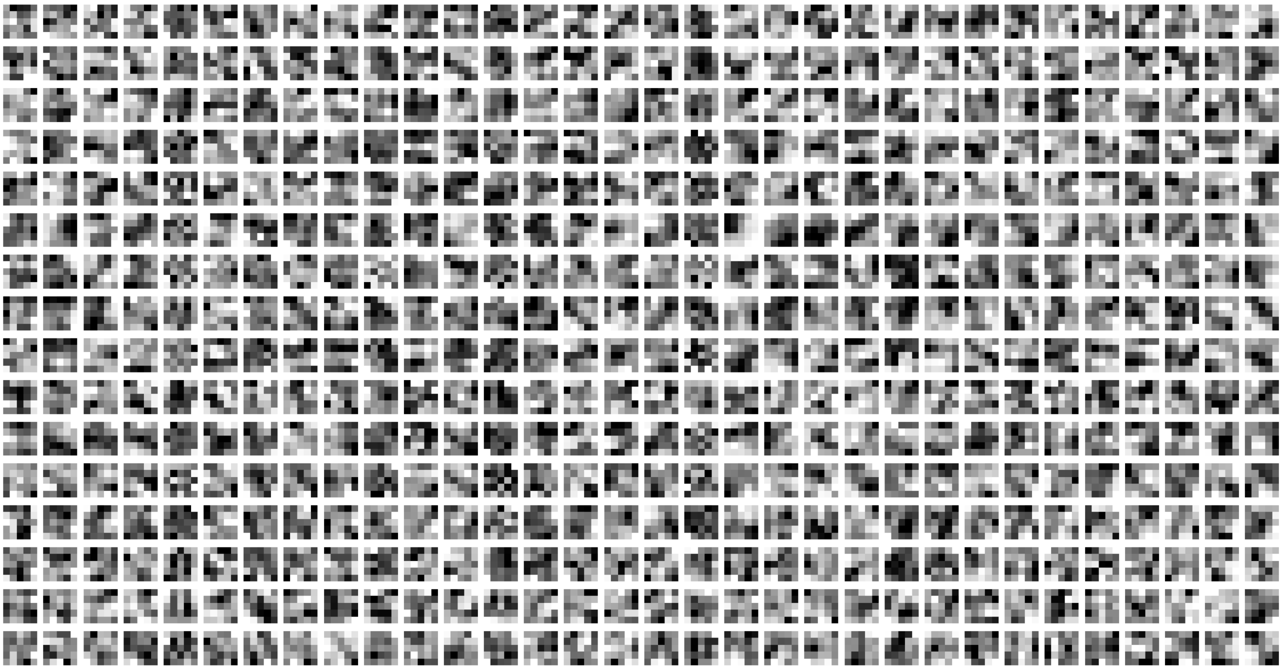

(h) DN2-TanH, third training replicate, second convolutional layer

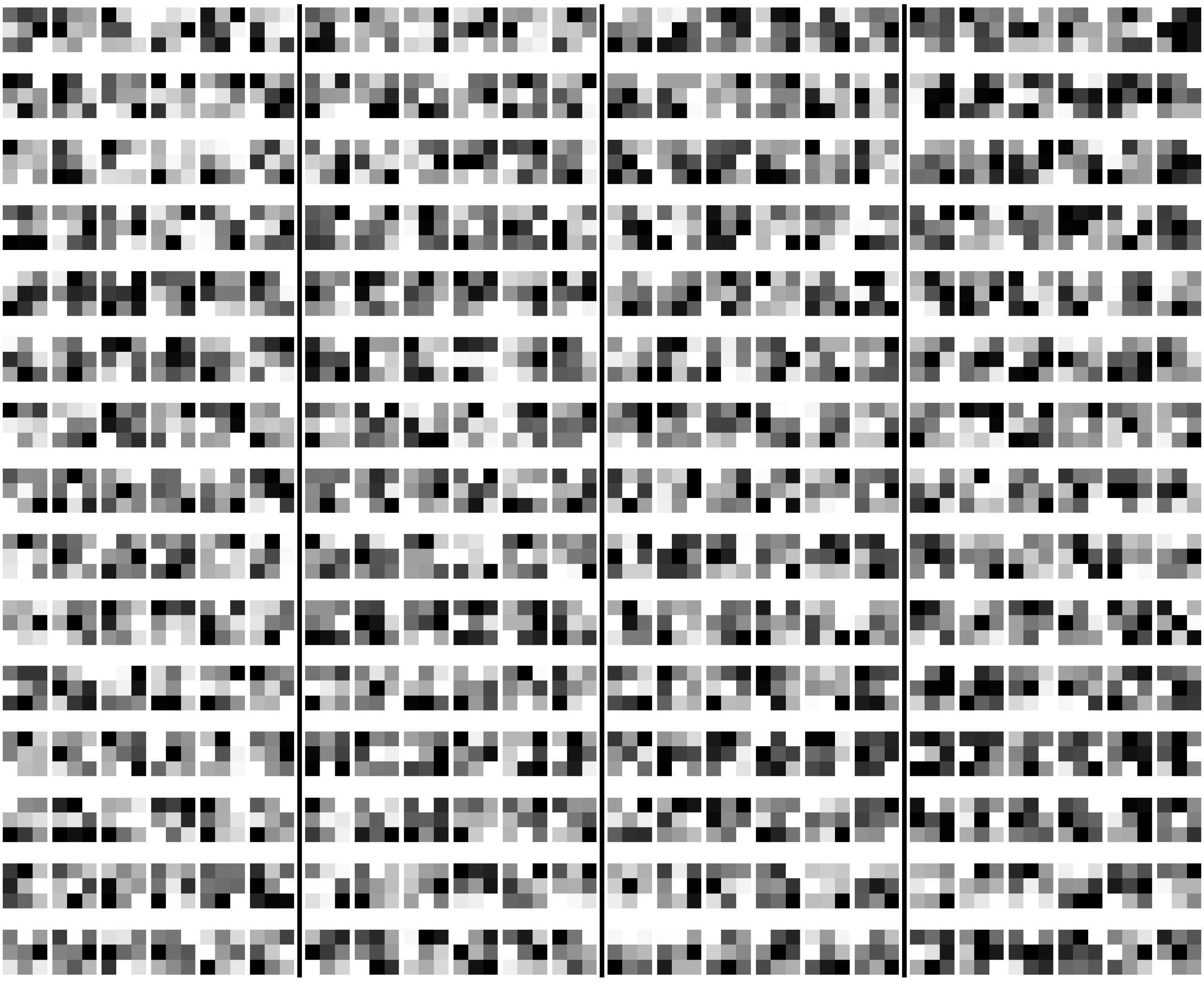

(i) DN2-TanH, third training replicate, third convolutional layer, 4 filter groups out of 32 shown

Figure S27. *Filters from the convolutional layers of the best deep CNN, DN2-TanH, for the three training replicates.*

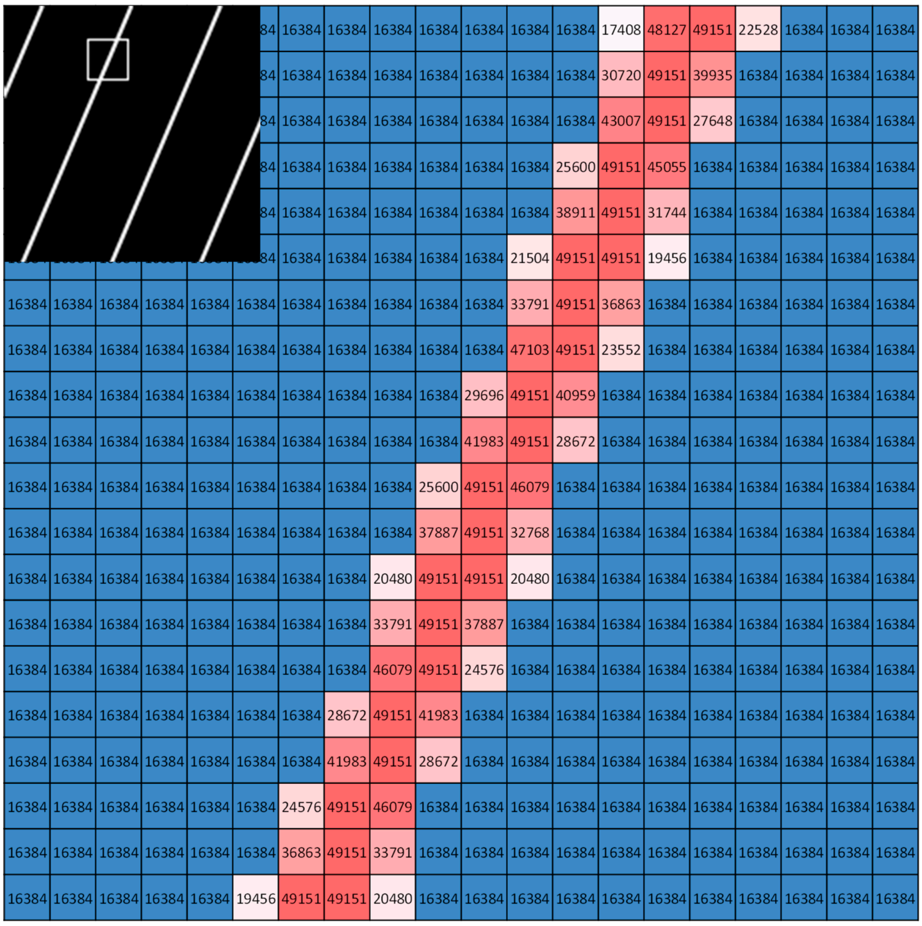

(a) bar of thickness 2 and angle of 157°

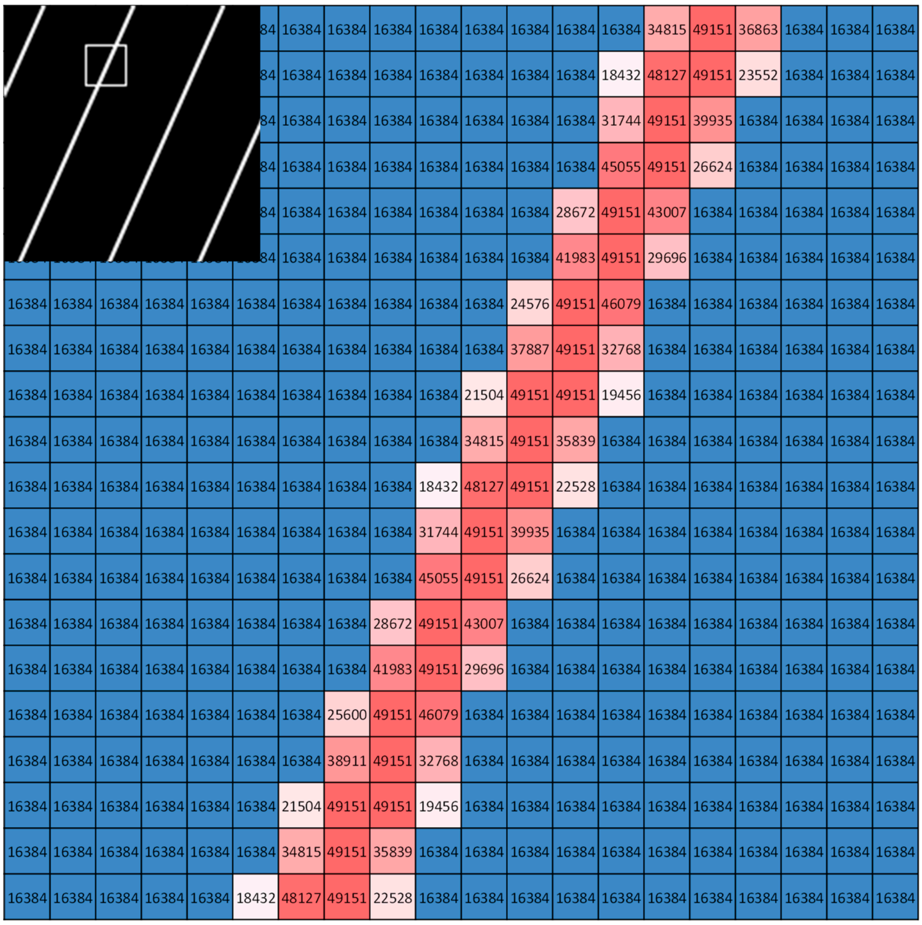

(b) bar of thickness 2 and angle of 156°

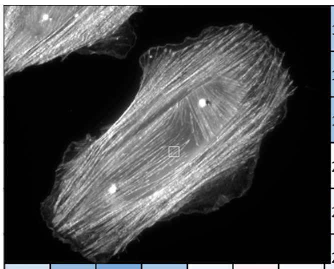

							1993	1978	1990	2216	2401	2233	2064	2059	1984	1981	1971	1898	1900
							1974	1936	2091	2379	2281	2155	2307	2325	2086	1941	1885	1916	2034
							1957	2100	2231	2182	2260	2586	2627	2139	1913	1919	1934	2071	2369
							2211	2196	2179	2374	2464	2430	2069	1877	1861	1994	2240	2424	2455
							2172	2108	2418	2369	2082	1941	1889	1951	2024	2522	2739	2340	2043
							2161	2124	2097	1980	1895	1924	1949	2134	2578	2685	2236	2001	1936
1950	1948	2050	2282	2297	2140	2108	2043	1891	1871	1852	1962	2133	2364	2577	2493	2160	1994	1963	1966
2106	2434	2445	2268	2230	2194	1960	1869	1842	1856	1908	2156	2773	2655	2252	2034	1944	1966	2030	2248
2657	2790	2440	2184	2188	1990	1867	1817	1819	1941	2287	2688	2709	2180	1977	2021	2114	2336	2598	2706
2673	2445	2298	2123	1926	1864	1849	1899	1968	2269	2619	2496	2116	2072	2028	2130	2635	3024	2925	2383
2403	2183	2013	1931	1886	1871	1959	2106	2308	2368	2270	2116	2072	2139	2227	2627	2951	2721	2270	2097
2132	1952	1850	1911	1928	2037	2327	2653	2498	2241	2160	2240	2329	2660	3099	3150	2594	2180	2083	2261
1918	1857	1878	1949	2142	2539	2913	2819	2321	2293	2533	2684	2964	3405	3467	2815	2094	2009	2236	2496
1899	1927	2027	2319	2784	2981	2799	2445	2409	2599	2764	2979	3047	3103	2646	2198	2039	2222	2534	2283
1987	2200	2546	2941	3076	2945	2712	2426	2408	2536	2815	2972	2714	2438	2253	2127	2211	2592	2475	2068
2311	2826	2911	2837	2987	2850	2516	2269	2345	2582	2880	2750	2302	2106	2112	2250	2528	2500	2141	1979
2796	2911	2823	2821	2672	2365	2235	2384	2538	2728	2680	2373	2105	2071	2190	2588	2701	2228	2040	2034
2725	2579	2707	2464	2252	2306	2586	2856	2920	2552	2327	2143	2103	2159	2555	2711	2308	2095	2068	2085
2564	2388	2287	2200	2338	2735	3136	2916	2440	2187	2179	2113	2129	2391	2753	2477	2148	2104	2085	2262
2355	2170	2217	2426	2642	2873	2836	2300	2159	2181	2128	2090	2284	2500	2320	2197	2128	2168	2313	2360

(c) actin fibers in fibroblast cell

Figure S28. *Instances of (a) and (b) synthetic textures and (c) real-life textures with complex signal. The insets show the whole images.*

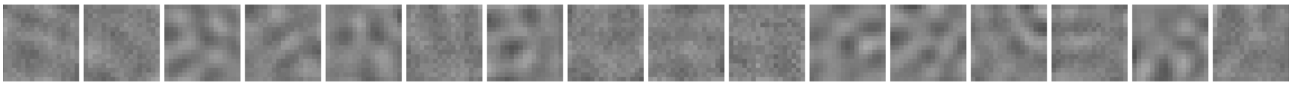

(a)

input texture	1st layer output	intensity std	intensity range
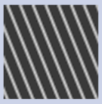	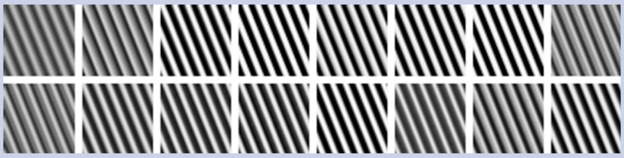	0.68	1.99
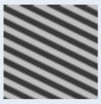	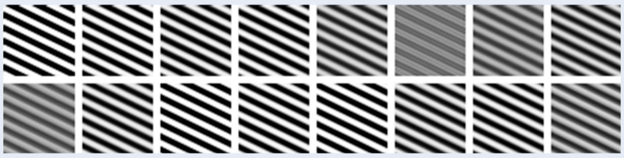	0.62	1.94
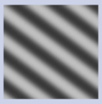	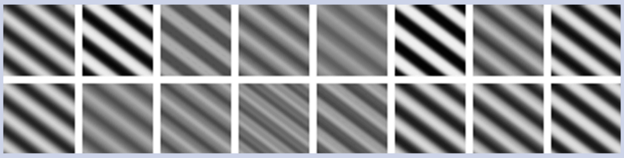	0.45	1.84
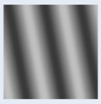	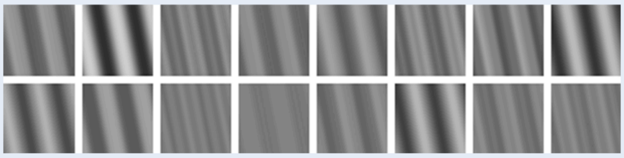	0.21	1.24
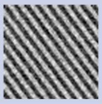	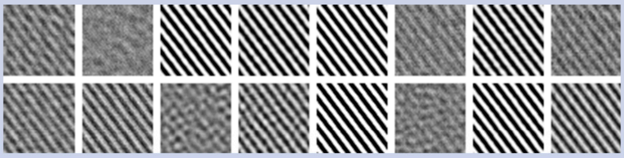	0.54	2.0
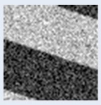	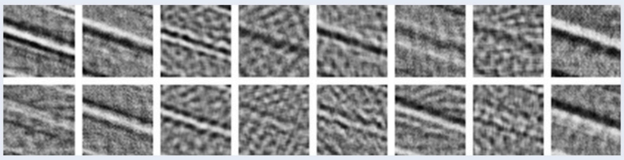	0.30	1.8

(b)

Figure S29 *Instances of convolutions of synthetic textures with kernels from the first layer of the deep network DN2. The figure shows: (a) 16 17x17 kernels; (b) from left to right: 6 synthetic textures with varying perturbation levels, for each synthetic texture the 16 outcomes of convolution followed by the TanH activation function using the kernels from (a), and the corresponding standard deviation and intensity range values.*

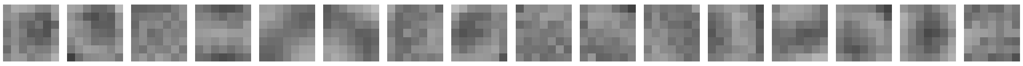

(a)

input texture	1st layer output	intensity std	intensity range
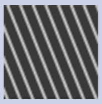	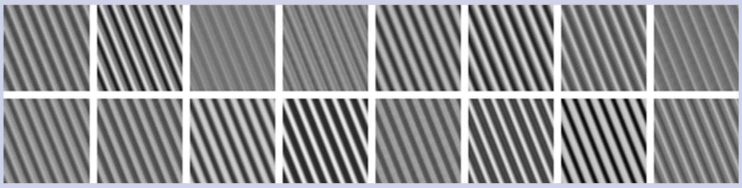	0.47	1.89
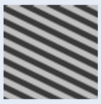	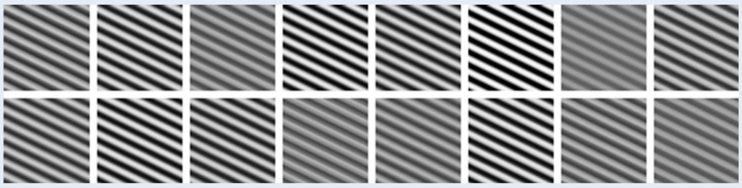	0.36	1.67
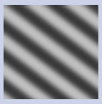	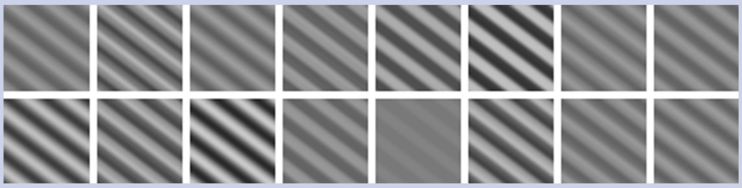	0.25	1.22
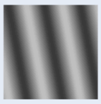	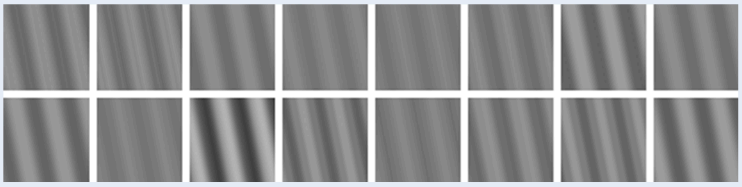	0.14	0,88
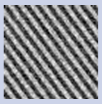	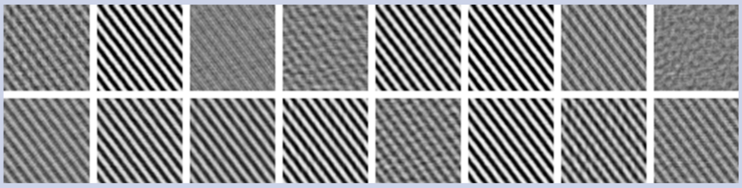	0.46	1.90
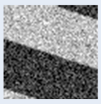	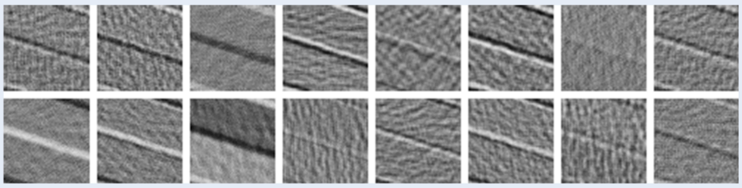	0.20	1.65

(b)

Figure S30. *Instances of convolutions of synthetic textures with kernels from the first layer of the deep network DN6. The figure shows: (a) 16 7x7 kernels; (b) from left to right: 6 synthetic textures with varying perturbation levels, for each synthetic texture the 16 outcomes of convolution followed by the TanH activation function using the kernels from (a), and the corresponding standard deviation and intensity range values.*

Table S1. Performance comparison between the best shallow and deep CNNs.

Gaussian noise std value	SN1-ELU RMSE (°)	DN2-TanH RMSE (°)
none	0.00	0.41
2000	0.01	0.39
4000	0.06	0.40
6000	0.23	0.46
8000	0.48	0.76

Blur kernel size	SN1-ELU RMSE (°)	DN2-TanH RMSE (°)
none	0.00	0.41
3x3	0.01	0.39
5x5	0.07	0.37
7x7	2.79	2.54
9x9	5.81	3.39

# tiles tested	SN1-ELU (tiles/s)	DN3-TanH (tiles/s)
178 605	6613.3	6821

Table S2. Directionality RMSE values vs. Gaussian noise standard deviation and averaging blur for deep networks DN2-TanH (first filter kernel 17x17) and DN6-TanH (first filter kernel 7x7)..

Gaussian noise std value	DN2-TanH RMSE (°)	DN12-TanH RMSE (°)
none	0.41	0.57
2000	0.39	0.53
4000	0.40	0.52
6000	0.46	0.57
8000	0.76	0.75

Blur kernel size	DN2-TanH RMSE (°)	DN12-TanH RMSE (°)
none	0.41	0.57
3x3	0.39	0.53
5x5	0.37	0.51
7x7	2.54	2.61
9x9	3.39	4.29
